# Genome-wide gene expression analysis for target genes to differentiate patients with intestinal tuberculosis and Crohn’s disease and discriminative value of FOXP3 mRNA expression

**DOI:** 10.1093/gastro/gov015

**Published:** 2015-05-11

**Authors:** Vineet Ahuja, Swati Subodh, Amit Tuteja, Veena Mishra, Sushil Kumar Garg, Neha Gupta, Govind Makharia, SK Acharya

**Affiliations:** ^1^Department of Gastroenterology and Human Nutrition, All India Institute of Medical Sciences, New Delhi, India, and; ^2^The Centre for Genomic Application (An IGIB–IMM collaboration), New Delhi, India

**Keywords:** Crohn’s disease, intestinal tuberculosis, microarray gene expression profiling, signaling pathway, FOXP3 mRNA

## Abstract

**Background and aims:** Crohn’s disease (CD) and intestinal tuberculosis (ITB) are both chronic granulomatous conditions with similar phenotypic presentations. Hence, there is need for a biomarker to differentiate between both these two diseases. This study aimed at genome-wide gene expression analysis of colonic biopsies from confirmed cases of ITB and CD in comparison with controls. To evaluate the role of T regulatory cells, forkhead box P3 (FOXP3) mRNA expression was quantified in serum as well as in colonic biopsies from patients with ITB and with the controls.

**Methods:** Paired samples, including serum and colonic biopsies, were taken from 33 study subjects (CD, ITB and controls), and total RNA was extracted. Human whole genome gene expression microarray analysis was performed using the Illumina HumanWG-6 BeadChip Kit with six total RNA samples of the three groups in duplicates. Real-time PCR for FOXP3 mRNA expression was analyzed in serum samples and colonic biopsy samples (4-CD, 5-ITB, 4-controls).

**Results:** In CD and ITB there was 1.5-fold upregulation of 92 and 382 genes and 1.5-fold downregulation of 91 and 256 genes, respectively. Peroxisome proliferators via the PPARγ pathway were most significantly downregulated (*P* < 0.005) in CD. Additionally, the IL4/5/6 signaling pathways and Toll-like receptor signaling pathway were identified as significantly differentially regulated (*P* < 0.005) at > 2-fold change. In ITB, the complement activation pathway, specifically the classical pathway, was the most significantly upregulated. FOXP3 mRNA expression was significantly elevated in colonic biopsies obtained from ITB patients as compared with CD cases (4.70 ± 2.21 *vs* 1.48 ± 0.31, *P* = 0.016).

**Conclusions:** FOXP3 mRNA expression in colonic mucosa could be a discriminatory marker between ITB and CD. Upregulation of the complement activation pathway in ITB suggests that pathogenetic mechanisms for ITB are similar to those of pulmonary tuberculosis. In CD, downregulation of PPARγ was seen in colonic tissue, suggesting that restoration of PPARγ-dependent anti-microbial barrier function may be a therapeutic target.

## Introduction

Intestinal tuberculosis (ITB) and Crohn’s disease (CD) are granulomatous diseases of the intestine. ITB is common in Asian countries, but CD is also being reported increasingly from all over Asia [1, 2]. The clinical, morphological and histological features of ITB and CD are so similar that it becomes difficult to differentiate between these two entities [3]. There is no definitive diagnostic test available to date for differentiating CD from ITB. There is also no single gold standard test for CD, and the diagnosis of CD is made on the basis of a combination of clinical, endoscopic, histological and radiological features. The diagnosis of ITB is made on the basis of presence of caseating granulomas and/or demonstration of acid-fast bacilli (AFB) by staining or culture and/or response to treatment with anti-tubercular therapy. Although demonstration of AFB is an important criterion for the diagnosis of ITB, the positivity rates of AFB vary from 10–30% only [4]. Therefore in a majority of patients with ITB, the initial diagnosis remains provisional, and the diagnosis is confirmed once the response to treatment is achieved. All of these situations highlight the need to establish the diagnosis of either CD or ITB before starting any form of empirical treatment.

Both ITB and CD occur due to excessive and inappropriate immunological response of the intestinal mucosa to an antigen/pathogen. While the pathogen in ITB cases is *Mycobacterium tuberculosis*, the pathogen/antigen in CD cases is still unidentified. Naturally occurring CD25 + CD4 + suppressor or T regulatory cells (Tregs) play an active part in establishing and maintaining immunological unresponsiveness to self-constituents (i.e. immunological self-tolerance) and negative control of various immune responses to nonself-antigens. Forkhead box P3 (FOXP3) is the intracellular marker of Tregs. Tregs have also been recently shown to suppress anti-microbial immune responses, especially against pathogens that establish persistent infections. In human tuberculosis, studies have shown increased numbers of Tregs in the blood and at sites of infection during active disease, but these studies were carried out in patients with pulmonary tuberculosis and not intestinal tuberculosis [5,6]. In auto-immune diseases like CD, there should be downregulation of Tregs, as seen in the few studies available [7,8].

Considering the different properties of Tregs, which include maintenance of immunological self-tolerance and negative control of pathological and physiological immune responses [9], suppression of anti-microbial immune responses and their increase in number in the blood and at site of infection during active disease, we planned to investigate for differential expression of Tregs in patients with ITB and CD. Conceivably, it may be possible to utilize FOXP3 expression levels as a differential marker between CD and ITB. There is no study that has looked at differential FOXP3 expression in serum and colonic mucosa in CD and ITB. Genome-wide expression analysis studies have been done for CD, yet there is no exploratory study available that has compared genome-wide gene expression in CD and ITB.

This study had two components. The first component explored the hypothesis-free aim of genome-wide gene expression analysis of mucosal target genes to differentiate between ITB and CD. The second component of the study was hypothesis driven, and the central theme was to evaluate the role of Tregs as a biomarker to differentiate between ITB and CD. Hence, FOXP3 mRNA expression was quantified by real-time PCR in serum as well as colonic biopsies of patients with CD, ITB and controls.

## Materials and Methods

### Human subjects

This was a cross-sectional study that included patients attending the Inflammatory Bowel Disease Clinic at All India Institute of Medical Sciences between January 2008 and May 2009. The patients were included in three groups: (i) ITB, (ii) CD and (iii) controls. Paired samples including serum and colonic biopsies were taken from each study subject. Colonic biopsies were taken during sigmoidoscopic/colonoscopic examinations from active lesions. ITB patients were included in the study before commencing anti-tubercular therapy. CD patients with a mild disease activity score between 150–300 were included in the study. The project was reviewed and approved by the Institute Ethics Committee. Informed consent was taken from each subject enrolled in this study. The laboratory investigators were blinded to the disease phenotype of the subjects and the control group.

The diagnosis of ITB was made on the basis of characteristic clinical features (abdominal pain, constipation and/or diarrhea, constitutional symptoms and intestinal obstruction), endoscopic features (ileocecal area involvement, ulcerations, nodularity and strictures), histological features (presence of granulomas) and micro-biological tests (presence of AFB on the smear examination or culture), and response to anti-tuberculous treatment (Paustian’s criteria with Logan’s modification) [10,11]. Only patients with ileocolonic tuberculosis were included in this study so as to maintain homogeneity of disease location.

The diagnosis of CD was established on the basis of the European Crohn’s and Colitis Organization guidelines, i.e. a combination of clinical, endoscopic and histological features [12]. Only patients with ileocolonic disease were included in this study to maintain homogeneity of disease location.

Controls were patients with suspected hemorrhoidal bleed who had been taken up for sigmoidoscopy.

### Patient sample collection

Paired samples (blood and colonic biopsy) were taken from the three study groups. Both samples were taken on the same day. Whole blood (3–5 ml) was collected in acid citrate dextrose vacutainers. Patients underwent colonoscopic examination using a video colonoscope after colon preparation with colonic lavage solution (polyethylene glycol). Multiple biopsies (10–15 mg of tissue) were taken from inflamed lesions during colonoscopy. The site of the biopsy was ileocolonic in both study groups. The biopsies were taken from sigmoid colon in control patients.

**Figure 1.**
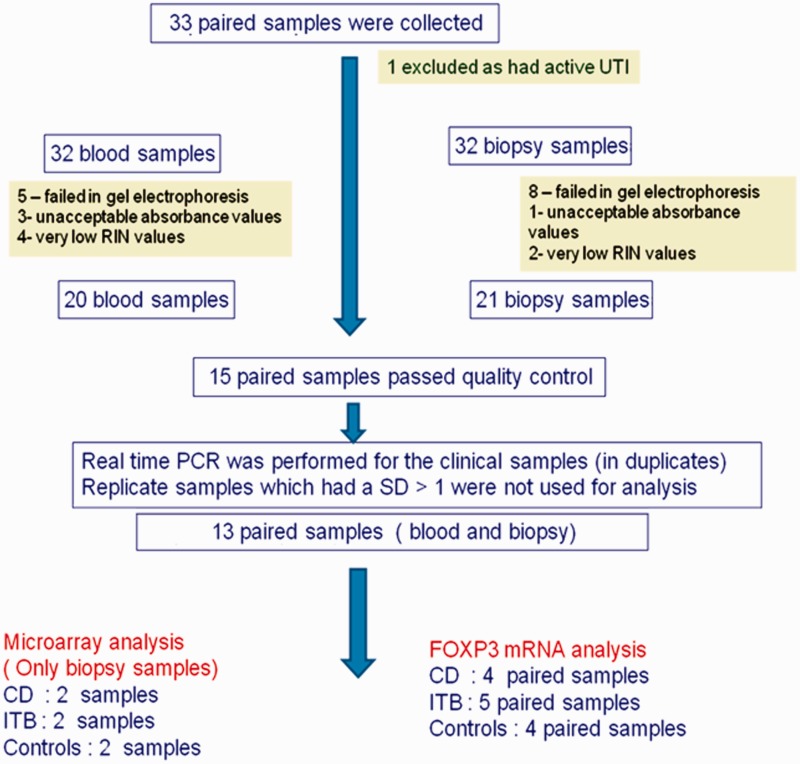
Flow chart showing sample acquisition (CD = Crohn’s disease; ITB = intestinal tuberculosis; UTI = urinary tract infection; RIN = RNA integrity number; SD = standard deviation).

A total of 33 patients were included in the study. After stringent quality checks for RNA integrity, 13 paired samples were available. These included five paired samples from ITB patients, four paired samples from CD patients and four paired samples from controls (Figure 1).

### RNA extraction and quality check

Blood samples were processed immediately, and biopsy samples were stored at −80°C until further processing for total RNA extraction. The Trizol (Invitrogen, USA) method was used for isolating RNA from blood within a few hours of receipt, whereas the RNeasy Mini Kit (Qiagen, USA) was used for total RNA isolation from biopsy samples as per the manufacturer’s instructions. The quality of the RNA was checked using the Agilent Bioanalyzer 2100 and then checked additionally with 1.2% denaturing formaldehyde gel.

### Microarray gene expression profiling

Illumina HumanWG-6 v3.0 Expression BeadChips (Illumina Inc.) were used for the microarray gene expression analysis of more than 48 000 human transcripts in each patient sample. These transcripts comprised 27 455 coding transcripts with well-established annotation, 7870 coding transcripts with provisional annotation, 446 non-coding transcripts with well-established annotation, 196 non-coding transcripts with provisional annotation and 12 837 experimentally confirmed mRNA sequences (candidate genes) that aligned to Expressed Sequence Tags (EST) clusters. The gene expression experiment was performed using the Illumina Human WG-6 v3 BeadChip kit as per the manufacturer’s instructions. A cutoff of 1.3-fold change was set to identify genes with significantly different expression. Genes with differential score > 13 (1.3 fold) and < −13 (−1.3 fold) were considered as upregulated genes and downregulated genes, respectively. The significant differential *P* values (*P* < 0.05), which had been analyzed using an Illumina custom model analogous to the *t* test error model, were accepted for further analysis. Further, the differentially expressed genes were subjected to multiple testing correction (Benajmini-Hochberg correction) to decrease the number of false positives. GOEAST (Gene Ontology Enrichment Analysis Software Toolkit) [13] and DAVID (Database for Annotation, Visualization and Integrated Discovery) [14,15] were used for predicting the gene ontology. BioCarta database search was performed for differentially regulated gene characterization (www.biocarta.com). Pathway miner BioRag (Bioresource for Array Genes at www.biorag.org) and DAVID were used to determine the significant pathway from differentially expressed genes based on their log ratios. The data discussed in this publication have been deposited in NCBI’s Gene Expression Omnibus [16] and are accessible through GEO Series accession number GSE26305 (http://www.ncbi.nlm.nih.gov/geo/query/acc.cgiacc=GSE26305).

### mRNA quantification of FOXP3 gene using real-time PCR

mRNA of the FOXP3 (target) gene was quantified with reference to GAPDH (reference) housekeeping gene using RT-qPCR. Primers used for FOXP3 gene amplification were 5’-CGGACCATCTTCTGGATGAG-3’ (forward primer) and 5’-TTGTCGGATGATGCCACAG-3’ (reverse primer). Primers for GAPDH amplification were 5’-TGTGGAAGGGCTCATGACCACAGTCCAT-3’ (forward primer) and 5’-GCCTGCTTCACCACCTTCTTGATG-3’ (reverse primer). SYBR Green chemistry was used for detection of the amplified fragment of the target and reference genes. The relative quantification of the target gene with respect to the housekeeping reference gene was done as described earlier [17]. REST (Relative Expression Software Tool) 2008 software was used for calculation of relative gene expression and statistical analysis [18].

### Statistical analysis

All data were expressed as mean ± standard deviation. Statistical significance was determined by Student *t* test for unpaired samples. For determining statistical significance of FOXP3 mRNA levels in ITB and CD patients due to the small sample size in each group, the data were mostly presented as median and interquartile range (IQR). Non-parametric analyses were used to calculate indicated *P* values and included a Mann-Whitney test (when comparing two unmatched samples). A *P* value < 0.05 was considered as significant.

## Results

Paired samples (blood and colonic biopsy) taken on the same day were studied from 13 subjects, which included four patients with CD, five with ITB and four controls. Clinical characteristics of the study groups are shown in **Table 1**.

**Table 1.** Study population characteristics

Characteristics	Crohn’s disease (*n* = 4)
Age, years	31 ± 9.8
Sex (male:female)	3:1
Disease duration, years	2.5 ± 91
Crohn’s disease activity index	190 ± 56
Phenotype	L3 B1 (ileocolonic inflammatory disease)
Past history of steroids	2 (50%)
Azathioprine/steroids in last three months	0
	Intestinal tuberculosis (*n* = 5)
Age, years	32.4 ± 6.9
Sex (male:female)	1:4
Disease extent	Ileocolonic 3; colonic 2
Associated Pulmonary Koch's	1(20%)
Caseating granulomas/ acid-fast bacilli	4(80%)
	Controls (*n* = 4)
Age, years	38.7 ± 10.1
Sex (male:female)	3:1
Indication for sigmoidoscopy	Hemorrhoidal bleed

### Differentially expressed genes in CD and ITB in comparison with normal controls

Microarray whole genome gene expression profile of the colonic biopsies was obtained from patients suffering with ITB (*n* = 2) and CD (*n* = 2) and compared with controls (*n* = 2). Within the same study group, the samples had a high degree of concordance (Pearson correlation coefficient >0.97), and thus no outliers were observed. Cluster analysis showed the samples of ITB to be grouped together, whereas the samples from CD and controls were clustered in different branches of the cluster (Supplementary Figure 1). A total of 13 374 genes were detected (*P* < 0.05) when the two patient groups were compared with the control group. In CD, of the 189 differentially expressed genes, 98 were upregulated and 91 were downregulated (*P* < 0.05). Of 638 genes that were differentially expressed in the ITB cases, 382 were upregulated, and 256 were downregulated (*P* < 0.05) (Supplementary Figure 2). Among these, a smaller subset of 112 and 561 genes was differentially expressing exclusively in CD and ITB cases, respectively (Supplementary Table 1). Seventy-six genes were found to be commonly differentially expressing in both patient groups in comparison with the controls. These comprised 50 downregulated and 26 upregulated genes (Supplementary Figure 3).

### Genes differentially expressed after multiple testing correction

After multiple testing correction, only two genes (CXCL6 and EGR1) were upregulated in CD. Their involvement has been reported in immunity, defense and signal transduction (www.biocarta.com). No downregulated genes were observed in comparison with the control group.

Seven genes (CCL13, COL4A1, DEFA5, FCN1, NPTX2, NR1H4 and REG1A) were upregulated in ITB. These genes were classified as immune and defense genes, structural genes, genes involved in wound healing and nucleic-acid binding. Two genes (GLDN and ISL1) were downregulated in ITB and were identified for their involvement in cell structure and mobility.

### Gene expression signatures as biomarkers for differentiating CD and ITB

**Figure 2.**
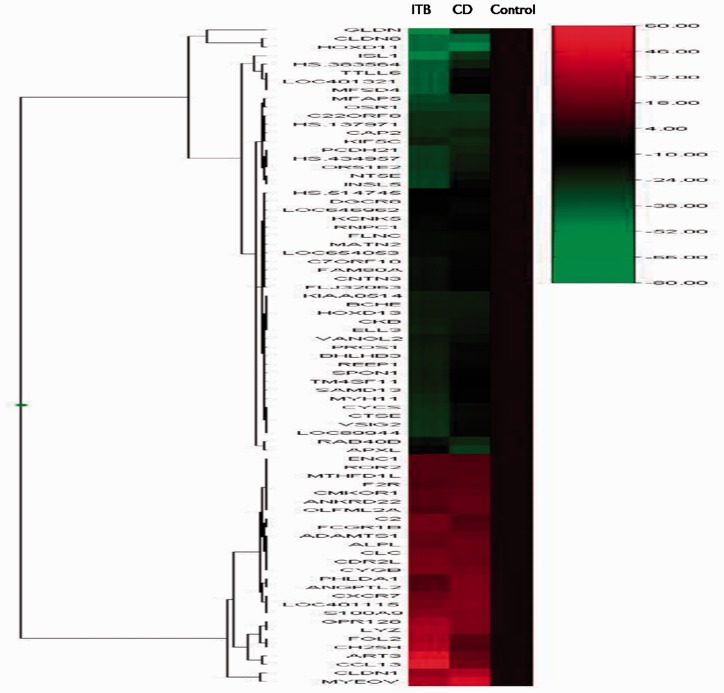
A heat map showing hierarchical clustering genes specifically regulated in patients with intestinal tuberculosis as compared with those with Crohn’s disease.

To investigate the gene expression pattern so as to identify candidate biomarkers for differentiating the two study groups, we went a step ahead to check for genes that are differentially regulating between CD and ITB only. We observed that there were 275 upregulated and 186 downregulated genes in ITB when compared with CD (Figure 2, Supplementary Table 2).

### Pathway analysis

#### Commonly regulating pathways

The pathways most significantly (*P* < 0.05) and substantially (>4 fold) regulated among the genes commonly differentially regulating in CD and ITB were the Irinotecan pathway, cell adhesion molecules and the complement pathway. *BChE* (butyrylcholinesterase) of the Irinotecan pathway was downregulated in both patient groups. There were no genes that showed upregulation in one and downregulation in the other within these 76 genes.

#### Differentially regulating pathways in CD patients

**Figure 3.**
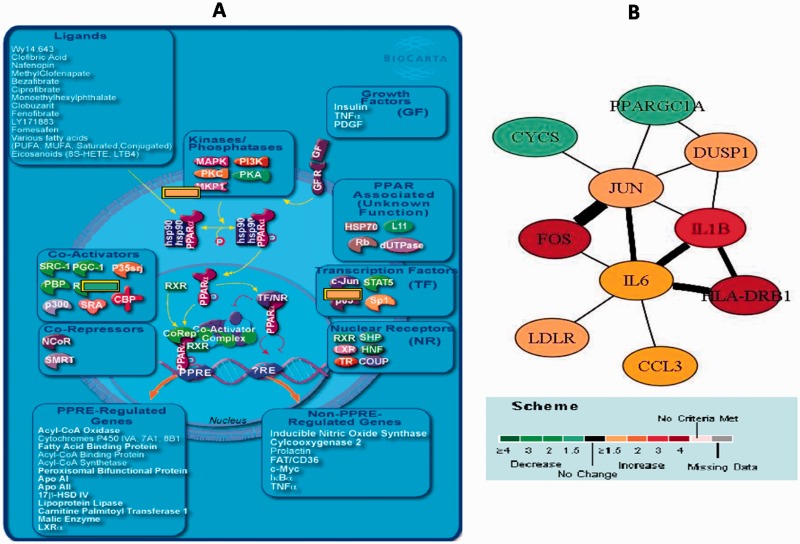
(**A**) Gene regulation by peroxisome proliferators (PPARs): the most significant pathway from Crohn’s disease (CD). Figure output from the Biocarta Database. (**B**) Biological network obtained from Pathway Miner at fold change =1.5 (Biocarta Database) for genes differentially regulated in the CD patients. The scale at the bottom of the figures shows the degree of regulation undergone by the individual genes. The thickness of the edges (connecting bars) depicts the number of pathways in which the respective genes are seen to interact. Greater thickness depicts gene interaction in more pathways.

Gene Ontology analysis, Biocarta and KEGG database search of the differentially regulated genes (>1.5 fold) revealed specific pathways associated with CD, of which the most significant (*P* < 0.005) was the pathway for mechanism of gene regulation by peroxisome proliferators via PPARγ (Figure 3A). Additionally, many signaling pathways, including the IL4/5/6 signaling pathways and Toll-like receptor signaling pathway, were identified as significantly differentially regulated (*P* < 0.005) at > 2-fold change. Besides the significance of individual genes (and gene products), it is essential to see how the differentially expressing genes interact with each other to form a network. To address this issue, we used Pathway Miner to analyze the networks that were significantly associated with the disease groups having component genes showing a ≥ 1.5-fold change. In CD, our pathway analysis suggested that the regulated genes were FOS, HLA-DRB1, IL1B, CCL3, JUN,DUSP1 and IL6, while the downregulated genes were PPARGC1A and GUC2B (Figure 3B). These genes were shown to be associated with each other in many different pathways including those found to be most significantly associated with CD in our study.

The level of GUC2B was elevated in colon as compared with other tissues; however, the levels were significantly decreased (>4 fold) in colonic biopsies of CD patients as compared with controls (*P* < 0.005). Another significantly downregulated gene (>1.5 fold) was the PPARGC1A, a peptide that is synthesized in the intestine at very low levels in the first place and further reduced in CD patients.

#### Differentially regulating pathways in ITB patients

**Figure 4.**
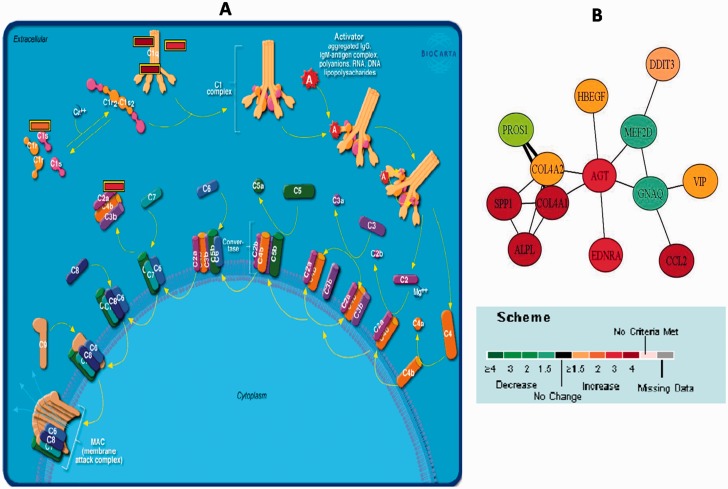
(**A**) Complement activation pathway: the most significant pathway from intestinal tuberculosis (ITB). Figure output from Biocarta Database. (**B**) Biological network obtained from Pathway Miner at fold change =1.5 (Biocarta Database) for genes differentially regulated in the ITB patients. The scale at the bottom of the figures shows the degree of regulation undergone by the individual genes. The thickness of the edges (connecting bars) depicts the number of pathways in which the respective genes are seen to interact. Greater thickness depicts gene interaction in more pathways.

The most significantly regulated pathway in ITB was the complement activation pathway, specifically the classical pathway (Figure 4A). The genes that were differentially expressing and part of this pathway were C1S, C1QC, SERPINA, VWF-2, C1QB and A2M. This pathway along with pathways involved in antigen processing and presentation (HLA-DMA, HLA-DPB1, CTSB, TAP1, HLA-DPA1, CD74, IF130, HLA-DRB5), smooth muscle contraction, cell-to-cell communication and cell adhesion were observed to be significantly (*P* < 0.001) and substantially (> 4 fold) regulated in ITB patients. Biological interaction network identified by Pathway Miner suggested that the genes upregulated in ITB were AGT, EDNRA, COL4A1, COL4A2, SSP1, CCL2 and ALPL, whereas, the downregulated genes were MEF2D and GNAQ (Figure 4 B).

#### FOXP3 mRNA quantification

**Figure 5.**
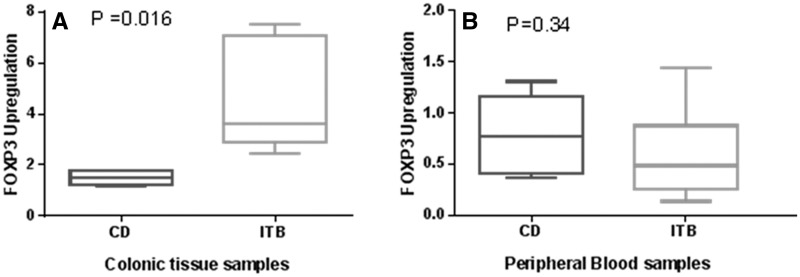
(**A**) Comparative FOXP3 mRNA expression levels in colonic biopsies from patients with Crohn’s disease and intestinal tuberculosis. **B)** Comparative FOXP3 mRNA expression levels in peripheral blood samples from patients with Crohn’s disease and intestinal tuberculosis.

The FOXP3 levels differed only marginally among the CD patients as compared with the controls in both serum and colonic biopsy (both *P* > 0.05). Among ITB patients, FOXP3 was marginally downregulated in the serum (*P* > 0.05), whereas it was markedly upregulated in the colonic biopsies (*P* < 0.05) as compared with the controls. We observed that the FOXP3 mRNA expression was significantly elevated in colonic biopsies (*P* < 0.05) obtained from ITB patients as compared with CD cases (4.70 ± 2.21 *vs* 1.48 ± 0.31, *P* = 0.016) (Figure 5A). No difference in FOXP3 mRNA expression was observed in the serum (*P* > 0.05) between ITB and CD patients (0.68 ± 0.58 *vs* 0.87 ± 0.42, *P* = 0.34) (Figure 5B). This implies that FOXP3 expression in colonic biopsies could be a discriminatory marker between ITB and CD.

## Discussion

Recent studies have identified the need and challenges of differentiating CD and ITB cases, especially in developing countries where ITB is endemic. Prior to a diagnosis of Crohn’s-specific cases, the first line of treatment given is generally for tuberculosis [2,3]. Clinical, histological and endoscopic parameters of analysis are not specific enough to provide a diagnosis of CD or ITB in a majority of cases, underscoring the search for candidate biomarkers for routine diagnostic application [1–3,19]. The current study compares gene expression in mucosal biopsies from patients with CD and ITB and highlights key pathways that are differentially regulated in both diseases. To date, this is the first study using gene expression to compare ITB and CD.

Pathway analysis of genome-wide gene expression profiles showed that FOS, HLA-DRB1, IL1B, CCL3, JUN, DUSP1 and IL6 were upregulated in CD, while PPARGC1A and GUC2B were downregulated. Of the pathways investigated, the peroxisome proliferator gene regulatory pathway was the most significantly downregulated in CD. In ITB, however, complement activation was the most significantly upregulated.

The key driving factors of intestinal inflammation are two stress-responsive transcription factors, AP-1 and NF-kB, which induce production of the inflammatory mediators TNF-α, IL-6, IL-12 and interferons [20–22]. AP-1 is a group of dimeric transcription factors comprising one of four Fos family proteins (c-Fos, FosB, Fra-1 and Fra-2) and one of three Jun family proteins (c-Jun, JunB and JunD). c-Fos is implicated in the regulation of innate immune responses. It has been reported that NF-kB activity is elevated in Fos-deficient mice, resulting in augmented inflammatory responses [23]. It has been shown that Fos proteins are negative regulators of NF-kβ mediated stress responses [24]. In our study, the *fos* gene significantly correlated with CD, suggesting that upregulation of *fos* in chronic intestinal inflammation may be a protective response. IL1B, another differentially regulated gene in our study, is produced by activated macrophages as a pro-protein, which is proteolytically processed into its active form by caspase 1 (CASP1/ICE). This cytokine is an important mediator of the inflammatory response and is involved in cell proliferation, differentiation and apoptosis. Another gene significantly upregulated in patients with CD was HLA-DRB1. In a study, the DRB3*0301/DRB1*1302 haplotype was the only significant MHC class II association noted in Caucasian people with CD and represented the strongest association of any MHC or non-MHC locus with this disease [25].

The nuclear receptor peroxisome proliferator-activated receptor-gamma (PPARγ) is expressed primarily in colonocytes. PPARγ is essential for intestinal homeostasis in response to both dietary- and microbiota-derived signals. PPARGC1A, which was downregulated in CD patients as compared with controls in this report, greatly increases the transcriptional activity of PPARγ. Upon recognition of either natural or synthetic ligands, formation of a heterodimer of retinoid X receptor alpha (RXRα) and PPARγ allows the regulation of a specific set of genes involved in intestinal homeostasis through its binding to PPARγ-response elements (PPREs) [26]. Genetic ablation of *PPARγ* has been reported to increase susceptibility to experimental colitis in rodents [27,28]. Other studies have shown that engagement of PPARγ-mediated signaling by its agonists such as rosiglitazone attenuated the severity of inflammatory lesions in both experimental and spontaneous models of colitis and might be effective in patients with ulcerative colitis [29]. The role of PPARγ in human colonic tissue in patients with CD has not been elucidated so far. We have shown that PPARγ is downregulated in colonic tissue in patients with CD when compared with normal controls. These findings support the development of PPARγ-targeting therapeutic and/or nutritional approaches to prevent colonic inflammation by restoring anti-microbial immunity in CD.

Here we report that various pathways such as the complement activation and coagulation cascades, antigen presenting and processing, and smooth muscle contraction play a significant role in patients with ITB. As no report is available on significantly upregulated pathways in ITB, we compared the gene expression analysis studies that are available for pulmonary tuberculosis [30]. This study revealed direct binding of human C1q to whole mycobacteria in the absence of antibodies [31]. In agreement with this observation, we found various classes of C1q genes upregulated. In addition, changes in the complement system have been reported in pulmonary tuberculosis. We found similar pathogenic mechanisms to be operative in ITB.

Interestingly, of the genes that are differentially regulated between CD and controls in the current study, only five of the upregulated genes (*ADM*, *ISG20, SPINK*4, *DSCR*1L and *STMN*2) have been previously identified [32]. Lack of concordance between different studies in terms of the genes, number of genes and their differential expression has been reported previously [32]. One reason for such variation could be the use of different platforms for the respective gene expression studies. Wu *et al.* used the Affymetrix GeneChip Human Genome U95Av2 arrays (12 625 probe sets for 9662 unique transcripts), and Costello *et al.* used a low-density cDNA array [32,33]. The numbers of gene transcripts that can be investigated in both cases were lower than the Illumina Human WG6 BeadChip (> 45 000 probes for almost 25 000 genes and candidate genes) used in the current study. Another reason for heterogeneity between the data could be the variation in stage of disease and the site from which the biopsies were collected in the studies. A limitation of our study is the relatively low sample number representative of the respective clinical profiles. The genes and pathways found to be most significantly affected in our study in the two disease groups need further validation in a larger set of patients. Our study, however, provides good indicators for the pathobiology of CD and ITB and could serve as a reference for large-scale studies.

FOXP3 T regulatory cells are required to prevent autoimmune disease but also prevent clearance of some chronic infections. Pulmonary tuberculosis is associated with increased FOXP3+ regulatory T cells at the site of active inflammation [34–36]. There are no data available on the FOXP3 mRNA expression in colonic tissues of patients with ITB. We hypothesized that FOXP3 mRNA expression should be different in ITB and CD at sites of active inflammation. To test this hypothesis, we performed a blinded study in which FOXP3 mRNA was quantified using quantitative real time PCR (qPCR) in paired (serum and colonic biopsies) samples from CD and ITB patients and compared with the control group. No difference was observed in FOXP3 gene expression in serum between the two disease groups, but ITB patients had statistically significantly higher expression of FOXP3 mRNA in colonic tissues as compared with CD, thereby supporting our hypothesis. The cost of the FOXP3 mRNA test in India is approximately equivalent to US$10. This test, once validated, would have immense application in differentiating between ITB and CD. Both diseases are phenotypically similar, and hence this test has the ability to assist physicians in resolving this clinical dilemma.

Interestingly, although FOXP3 did not appear among the differentially expressing genes in the whole genome expression profiling, it was significantly overexpressed in the colonic biopsies of ITB as compared with CD patients. This observation is not a discrepancy in our results; rather it is the outcome of different technology platforms. The whole genome expression profiling was done by microarrays, which are a hybridization-based technology, whereby specific probes bind to the mRNA-derived cDNA from the samples. The signal intensity generated as a result of this hybridization is detected and quantified. Firstly, low abundant transcripts or low expressing genes may not be detected at significant levels within the threshold parameters due to their low signal intensities. This would convey the absence of these genes in the sample being studied. On the other hand, mRNA quantification was evaluated using qPCR, in which low initial sample can be amplified and detected within the parameters of detection and quantification. Second, the Illumina WG6 array does not have probes for identifying the different isoforms of FOXP3 gene. In such cases, an isoform arising due to alternative splicing will not be detected in microarrays. In contrast, the primers designed for FOXP3 detection were designed on exons 5 and 8, which are present in the FOXP3 transcript irrespective of the isoform.

In conclusion, FOXP3 mRNA expression could be a discriminatory marker between ITB and CD. Other target genes (JUN, FOS, ILIB, HLADRB1, IL6 and CCL3) identified by our study need further validation to evaluate their utility as differentiating biomarkers.

## Funding

This project was undertaken under the ‘FIST' scheme of Department of Science and Technology, Government of India. In addition, support was taken from ICMR Senior Research Fellowship granted to VM.

## Supplementary Data

Supplementary data are available at *Gastroenterology Report* online.

*Conflict of interest statement:* none declared.

Supplementary Data
