# Supplementary material for: Genome-wide gene expression analysis for target genes to differentiate patients with intestinal tuberculosis and Crohn’s disease and discriminative value of FOXP3 mRNA expression
Source: Gastroenterol Rep (Oxf). 2015 May 11;4(1):59–67. doi: 10.1093/gastro/gov015 (PMC4760064; doi:10.1093/gastro/gov015)
Supplement: Supplementary Data [file supp_4_1_59__index.html]

Genome-wide gene expression analysis for target genes to differentiate patients with intestinal tuberculosis and Crohn’s disease and discriminative value of FOXP3 mRNA expression — Genome-wide gene expression analysis for target genes to differentiate patients with intestinal tuberculosis and Crohn’s disease and discriminative value of FOXP3 mRNA expression — Genome-wide gene expression analysis for target genes to differentiate patients with intestinal tuberculosis and Crohn’s disease and discriminative value of FOXP3 mRNA expression — Supplementary Data 

# Genome-wide gene expression analysis for target genes to differentiate patients with intestinal tuberculosis and Crohn’s disease and discriminative value of FOXP3 mRNA expression

## Supplementary Data

files

**Files in this Data Supplement:**

- Supplementary Data - zip file
