# Supplementary figures and images for: Genome-wide gene expression analysis for target genes to differentiate patients with intestinal tuberculosis and Crohn’s disease and discriminative value of FOXP3 mRNA expression
Source: Gastroenterol Rep (Oxf). 2015 May 11;4(1):59–67. doi: 10.1093/gastro/gov015 (PMC4760064; doi:10.1093/gastro/gov015)

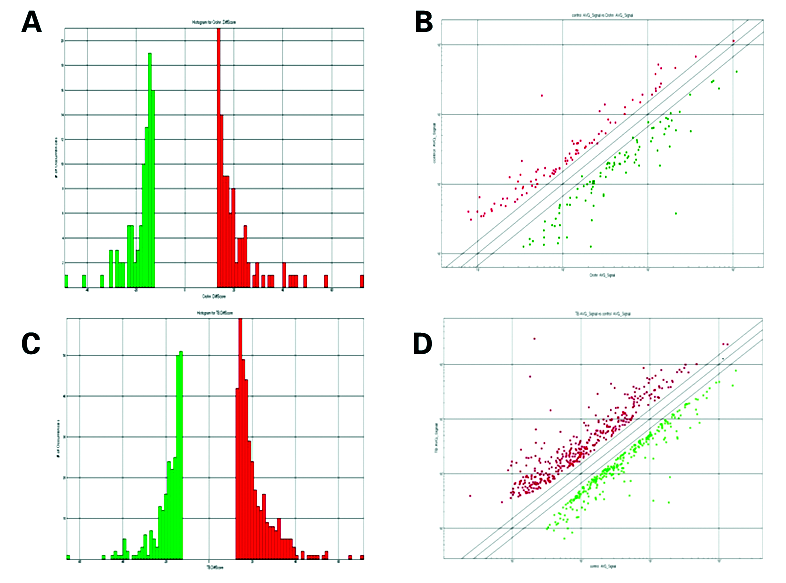

Supplement: Supplementary Data [file supp_gov015_suppl_data.zip › Supplementary Figure 2.TIF]

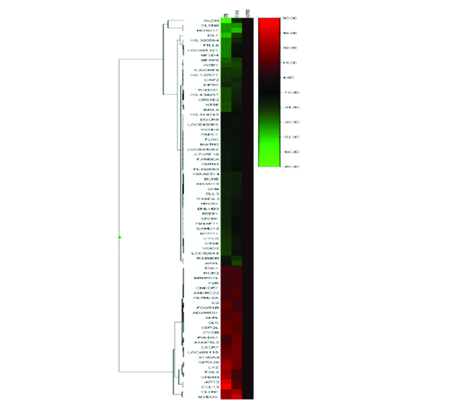

Supplement: Supplementary Data [file supp_gov015_suppl_data.zip › Supplementary Figure 3.TIF]

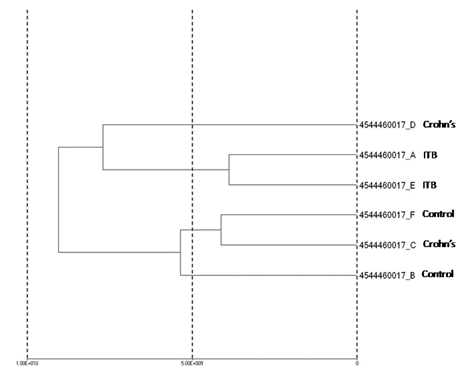

Supplement: Supplementary Data [file supp_gov015_suppl_data.zip › Supplementary Figure 1.TIF]
