# Supplementary material for: Genome-wide gene expression analysis for target genes to differentiate patients with intestinal tuberculosis and Crohn’s disease and discriminative value of FOXP3 mRNA expression
Source: Gastroenterol Rep (Oxf). 2015 May 11;4(1):59–67. doi: 10.1093/gastro/gov015 (PMC4760064; doi:10.1093/gastro/gov015)
Supplement: Supplementary Data [file supp_gov015_suppl_data.zip › Supplementary_Table_1.doc]

| ***Crohn’s Disease*** | | | | |
| --- | --- | --- | --- | --- |
| **Gene Symbol** | | **Description** | | **Regulation** |
| NGFRAP1L1 | | Homo sapiens NGFRAP1-like 1 (NGFRAP1L1), mRNA. | | Down regulated |
| LOC63928 | | Homo sapiens hepatocellular carcinoma antigen gene 520 (LOC63928), mRNA. | | Down regulated |
| FEV | | Homo sapiens FEV (ETS oncogene family) (FEV), mRNA. | | Down regulated |
| KLRC2 | | Homo sapiens killer cell lectin-like receptor subfamily C, member 2 (KLRC2), mRNA. | | Down regulated |
| FLJ16369 | | PREDICTED: Homo sapiens similar to RIKEN cDNA 5830406J20 (FLJ16369), mRNA. | | Down regulated |
| CRIP1 | | Homo sapiens cysteine-rich protein 1 (intestinal) (CRIP1), mRNA. | | Down regulated |
| TTR | | Homo sapiens transthyretin (prealbumin, amyloidosis type I) (TTR), mRNA. | | Down regulated |
| EDIL3 | | Homo sapiens EGF-like repeats and discoidin I-like domains 3 (EDIL3), mRNA. | | Down regulated |
| LOC649210 | | PREDICTED: Homo sapiens similar to Ig lambda chain V region 4A precursor (LOC649210), mRNA. | | Down regulated |
| HS.445274 | | DKFZp313P1922_r1 313 (synonym: hlcc2) Homo sapiens cDNA clone DKFZp313P1922 5, mRNA sequence | | Down regulated |
| NAALADL1 | | Homo sapiens N-acetylated alpha-linked acidic dipeptidase-like 1 (NAALADL1), mRNA. | | Down regulated |
| SLC6A12 | | Homo sapiens solute carrier family 6 (neurotransmitter transporter, betaine/GABA), member 12 (SLC6A12), mRNA. | | Down regulated |
| HOXB13 | | Homo sapiens homeo box B13 (HOXB13), mRNA. | | Down regulated |
| HS.134650 | | AGENCOURT_13567612 NIH_MGC_184 Homo sapiens cDNA clone IMAGE:30326575 5, mRNA sequence | | Down regulated |
| TNFAIP8L3 | | Homo sapiens tumor necrosis factor, alpha-induced protein 8-like 3 (TNFAIP8L3), mRNA. | | Down regulated |
| CYP27A1 | | Homo sapiens cytochrome P450, family 27, subfamily A, polypeptide 1 (CYP27A1), nuclear gene encoding mitochondrial protein, mRNA. | | Down regulated |
| GUCA2B | | Homo sapiens guanylate cyclase activator 2B (uroguanylin) (GUCA2B), mRNA. | | Down regulated |
| PPP1R3C | | Homo sapiens protein phosphatase 1, regulatory (inhibitor) subunit 3C (PPP1R3C), mRNA. | | Down regulated |
| FLJ23436 | | Homo sapiens hypothetical protein FLJ23436 (FLJ23436), mRNA. | | Down regulated |
| LOC645719 | | PREDICTED: Homo sapiens similar to ciliary rootlet coiled-coil, rootletin (LOC645719), mRNA. | | Down regulated |
| C6ORF160 | | PREDICTED: Homo sapiens chromosome 6 open reading frame 160, transcript variant 4 (C6orf160), mRNA. | | Down regulated |
| FREQ | | Homo sapiens frequenin homolog (Drosophila) (FREQ), mRNA. | | Down regulated |
| ZNF680 | | Homo sapiens zinc finger protein 680 (ZNF680), mRNA. | | Down regulated |
| ZNF291 | | Homo sapiens zinc finger protein 291 (ZNF291), mRNA. | | Down regulated |
| LOC649518 | | PREDICTED: Homo sapiens similar to 40S ribosomal protein S26 (LOC649518), mRNA. | | Down regulated |
| C14ORF122 | | Homo sapiens chromosome 14 open reading frame 122 (C14orf122), mRNA. | | Down regulated |
| C9ORF123 | | Homo sapiens chromosome 9 open reading frame 123 (C9orf123), mRNA. | | Down regulated |
| LOC650298 | | PREDICTED: Homo sapiens similar to 40S ribosomal protein S26 (LOC650298), mRNA. | | Down regulated |
| SCG2 | | Homo sapiens secretogranin II (chromogranin C) (SCG2), mRNA. | | Down regulated |
| RPS26L | | Homo sapiens 40S ribosomal protein S26-like (RPS26L) on chromosome 13. | | Down regulated |
| NPDC1 | | Homo sapiens neural proliferation, differentiation and control, 1 (NPDC1), mRNA. | | Down regulated |
| PAG1 | | Homo sapiens phosphoprotein associated with glycosphingolipid microdomains 1 (PAG1), mRNA. | | Down regulated |
| LOC644191 | | PREDICTED: Homo sapiens similar to 40S ribosomal protein S26, transcript variant 2 (LOC644191), mRNA. | | Down regulated |
| PROM2 | | Homo sapiens prominin 2 (PROM2), mRNA. | | Down regulated |
| CPE | | Homo sapiens carboxypeptidase E (CPE), mRNA. | | Down regulated |
| PPARGC1A | | Homo sapiens peroxisome proliferative activated receptor, gamma, coactivator 1, alpha (PPARGC1A), mRNA. | | Down regulated |
| LOC643516 | | PREDICTED: Homo sapiens similar to 40S ribosomal protein S26 (LOC643516), mRNA. | | Down regulated |
| ZMYM1 | | Homo sapiens zinc finger, MYM-type 1 (ZMYM1), mRNA. | | Down regulated |
| HSD11B2 | | Homo sapiens hydroxysteroid (11-beta) dehydrogenase 2 (HSD11B2), mRNA. | | Down regulated |
| DENND4C | | Homo sapiens DENN/MADD domain containing 4C (DENND4C), mRNA. | | Down regulated |
| BTG3 | | Homo sapiens BTG family, member 3 (BTG3), mRNA. | | Down regulated |
| LOC388743 | | PREDICTED: Homo sapiens similar to calpain 8, transcript variant 5 (LOC388743), mRNA. | | up regulated |
| SLC38A1 | | Homo sapiens solute carrier family 38, member 1 (SLC38A1), mRNA. | | up regulated |
| MBP | | Homo sapiens myelin basic protein (MBP), transcript variant 7, mRNA. | | up regulated |
| LDLR | | Homo sapiens low density lipoprotein receptor (familial hypercholesterolemia) (LDLR), mRNA. | | up regulated |
| BCL10 | | Homo sapiens B-cell CLL/lymphoma 10 (BCL10), mRNA. | | up regulated |
| RASD2 | | Homo sapiens RASD family, member 2 (RASD2), mRNA. | | up regulated |
| DLL1 | | Homo sapiens delta-like 1 (Drosophila) (DLL1), mRNA. | | up regulated |
| TAPBPL | | Homo sapiens TAP binding protein-like (TAPBPL), mRNA. | | up regulated |
| LOC654174 | | PREDICTED: Homo sapiens similar to CG4775-PA (LOC654174), mRNA. | | up regulated |
| BAIAP2L1 | | Homo sapiens BAI1-associated protein 2-like 1 (BAIAP2L1), mRNA. | | up regulated |
| SLC7A1 | | Homo sapiens solute carrier family 7 (cationic amino acid transporter, y+ system), member 1 (SLC7A1), mRNA. | | up regulated |
| JUN | | Homo sapiens jun oncogene (JUN), mRNA. | | up regulated |
| DNAJC12 | | Homo sapiens DnaJ (Hsp40) homolog, subfamily C, member 12 (DNAJC12), transcript variant 2, mRNA. | | up regulated |
| FUT2 | | Homo sapiens fucosyltransferase 2 (secretor status included) (FUT2), mRNA. | | up regulated |
| CBS | | Homo sapiens cystathionine-beta-synthase (CBS), mRNA. | | up regulated |
| SERPINB8 | | Homo sapiens serpin peptidase inhibitor, clade B (ovalbumin), member 8 (SERPINB8), transcript variant 3, mRNA. | | up regulated |
| NQO2 | | Homo sapiens NAD(P)H dehydrogenase, quinone 2 (NQO2), mRNA. | | up regulated |
| STMN2 | | Homo sapiens stathmin-like 2 (STMN2), mRNA. | | up regulated |
| TM4SF1 | | Homo sapiens transmembrane 4 L six family member 1 (TM4SF1), mRNA. | | up regulated |
| ISG20 | | Homo sapiens interferon stimulated exonuclease gene 20kDa (ISG20), mRNA. | | up regulated |
| DSCR1 | | Homo sapiens Down syndrome critical region gene 1 (DSCR1), transcript variant 2, mRNA. | | up regulated |
| LOC201175 | | Homo sapiens hypothetical protein LOC201175 (LOC201175), mRNA. | | up regulated |
| DUSP1 | | Homo sapiens dual specificity phosphatase 1 (DUSP1), mRNA. | | up regulated |
| FAM57A | | Homo sapiens family with sequence similarity 57, member A (FAM57A), mRNA. | | up regulated |
| PSTPIP2 | | Homo sapiens proline-serine-threonine phosphatase interacting protein 2 (PSTPIP2), mRNA. | | up regulated |
| LOC651441 | | PREDICTED: Homo sapiens similar to Fc fragment of IgG binding protein (LOC651441), mRNA. | | up regulated |
| MICB | | Homo sapiens MHC class I polypeptide-related sequence B (MICB), mRNA. | | up regulated |
| NCOA7 | | Homo sapiens nuclear receptor coactivator 7 (NCOA7), mRNA. | | up regulated |
| ADM2 | | Homo sapiens adrenomedullin 2 (ADM2), mRNA. | | up regulated |
| LAMC3 | | Homo sapiens laminin, gamma 3 (LAMC3), mRNA. | | up regulated |
| F3 | | Homo sapiens coagulation factor III (thromboplastin, tissue factor) (F3), mRNA. | | up regulated |
| CCRN4L | | Homo sapiens CCR4 carbon catabolite repression 4-like (S. cerevisiae) (CCRN4L), mRNA. | | up regulated |
| PLAUR | | Homo sapiens plasminogen activator, urokinase receptor (PLAUR), transcript variant 2, mRNA. | | up regulated |
| ACP5 | | Homo sapiens acid phosphatase 5, tartrate resistant (ACP5), mRNA. | | up regulated |
| ACE2 | | Homo sapiens angiotensin I converting enzyme (peptidyl-dipeptidase A) 2 (ACE2), mRNA. | | up regulated |
| MTNR1A | | Homo sapiens melatonin receptor 1A (MTNR1A), mRNA. | | up regulated |
| PXDN | | Homo sapiens peroxidasin homolog (Drosophila) (PXDN), mRNA. XM_935184 XM_936786 XM_942966 XM_942982 XM_942986 XM_942994 XM_943000 | | up regulated |
| C12ORF36 | | Homo sapiens chromosome 12 open reading frame 36 (C12orf36), mRNA. | | up regulated |
| IGSF4 | | Homo sapiens cell adhesion molecule 1 (CADM1), mRNA. | | up regulated |
| ERRFI1 | | Homo sapiens ERBB receptor feedback inhibitor 1 (ERRFI1), mRNA. | | up regulated |
| LOC652332 | | PREDICTED: Homo sapiens similar to mixed lineage kinase domain-like (LOC652332), mRNA. | | up regulated |
| ZBP1 | | Homo sapiens Z-DNA binding protein 1 (ZBP1), mRNA. | | up regulated |
| IRS2 | | Homo sapiens insulin receptor substrate 2 (IRS2), mRNA. | | up regulated |
| CYP2C18 | | Homo sapiens cytochrome P450, family 2, subfamily C, polypeptide 18 (CYP2C18), mRNA. | | up regulated |
| CTGF | | Homo sapiens connective tissue growth factor (CTGF), mRNA. | | up regulated |
| CHRM3 | | Homo sapiens cholinergic receptor, muscarinic 3 (CHRM3), mRNA. | | up regulated |
| IER3 | | Homo sapiens immediate early response 3 (IER3), transcript variant long, mRNA. | | up regulated |
| ASNS | | Homo sapiens asparagine synthetase (ASNS), transcript variant 1, mRNA. | | up regulated |
| SPINK4 | | Homo sapiens serine peptidase inhibitor, Kazal type 4 (SPINK4), mRNA. | | up regulated |
| LOC648470 | | PREDICTED: Homo sapiens similar to Caspase-4 precursor (CASP-4) (ICH-2 protease) (TX protease) (ICE(rel)-II) (LOC648470), mRNA. | | up regulated |
| AZGP1 | | Homo sapiens alpha-2-glycoprotein 1, zinc-binding (AZGP1), mRNA. | | up regulated |
| APOL6 | | Homo sapiens apolipoprotein L, 6 (APOL6), mRNA. | | up regulated |
| REG4 | | Homo sapiens regenerating islet-derived family, member 4 (REG4), mRNA. | | up regulated |
| IL6 | | Homo sapiens interleukin 6 (interferon, beta 2) (IL6), mRNA. | | up regulated |
| TRIM31 | | Homo sapiens tripartite motif-containing 31 (TRIM31), mRNA. | | up regulated |
| LOC401074 | | PREDICTED: Homo sapiens hypothetical LOC401074 (LOC401074), mRNA. | | up regulated |
| CCL3 | | Homo sapiens chemokine (C-C motif) ligand 3 (CCL3), mRNA. | | up regulated |
| C8ORF4 | | Homo sapiens chromosome 8 open reading frame 4 (C8orf4), mRNA. | | up regulated |
| IL8 | | Homo sapiens interleukin 8 (IL8), mRNA. | | up regulated |
| ANGPTL4 | | Homo sapiens angiopoietin-like 4 (ANGPTL4), transcript variant 1, mRNA. | | up regulated |
| NR4A2 | | Homo sapiens nuclear receptor subfamily 4, group A, member 2 (NR4A2), transcript variant 1, mRNA. | | up regulated |
| PHGDH | | Homo sapiens phosphoglycerate dehydrogenase (PHGDH), mRNA. | | up regulated |
| IL1B | | Homo sapiens interleukin 1, beta (IL1B), mRNA. | | up regulated |
| SLC7A5 | | Homo sapiens solute carrier family 7 (cationic amino acid transporter, y+ system), member 5 (SLC7A5), mRNA. | | up regulated |
| TMSB4Y | | Homo sapiens thymosin, beta 4, Y-linked (TMSB4Y), mRNA. | | up regulated |
| FOS | | Homo sapiens v-fos FBJ murine osteosarcoma viral oncogene homolog (FOS), mRNA. | | up regulated |
| KRT6B | | Homo sapiens keratin 6B (KRT6B), mRNA. | | up regulated |
| FOSB | | Homo sapiens FBJ murine osteosarcoma viral oncogene homolog B (FOSB), mRNA. | | up regulated |
| EGR1 | | Homo sapiens early growth response 1 (EGR1), mRNA. | | up regulated |
| CXCL6 | | Homo sapiens chemokine (C-X-C motif) ligand 6 (granulocyte chemotactic protein 2) (CXCL6), mRNA. | | up regulated |
| HLA-DRB1 | | Homo sapiens major histocompatibility complex, class II, DR beta 1 (HLA-DRB1), mRNA. | | up regulated |
|  | ***Intestinal tuberculosis*** | |  | |
| **Gene Symbol** | **Description** | | **Regulation** | |
| CAPN13 | Homo sapiens calpain 13 (CAPN13), mRNA. | | Down regulated | |
| C14ORF29 | Homo sapiens abhydrolase domain containing 12B (ABHD12B), transcript variant 2, mRNA. | | Down regulated | |
| SLC28A2 | Homo sapiens solute carrier family 28 (sodium-coupled nucleoside transporter), member 2 (SLC28A2), mRNA. | | Down regulated | |
| CD177 | Homo sapiens CD177 antigen (CD177), mRNA. | | Down regulated | |
| SPINK5 | Homo sapiens serine peptidase inhibitor, Kazal type 5 (SPINK5), mRNA. | | Down regulated | |
| ST6GALNAC6 | Homo sapiens ST6 (alpha-N-acetyl-neuraminyl-2,3-beta-galactosyl-1, 3)-N-acetylgalactosaminide alpha-2,6-sialyltransferase 6 (ST6GALNAC6), mRNA. | | Down regulated | |
| DES | Homo sapiens desmin (DES), mRNA. | | Down regulated | |
| HS.437111 | AGENCOURT_6796899 NIH_MGC_85 Homo sapiens cDNA clone IMAGE:5787825 5, mRNA sequence | | Down regulated | |
| SLPI | Homo sapiens secretory leukocyte peptidase inhibitor (SLPI), mRNA. | | Down regulated | |
| CYP4F11 | Homo sapiens cytochrome P450, family 4, subfamily F, polypeptide 11 (CYP4F11), mRNA. | | Down regulated | |
| CNN1 | Homo sapiens calponin 1, basic, smooth muscle (CNN1), mRNA. | | Down regulated | |
| LOC647987 | PREDICTED: Homo sapiens similar to Ribosome biogenesis protein BMS1 homolog (LOC647987), mRNA. | | Down regulated | |
| KLK15 | Homo sapiens kallikrein-related peptidase 15 (KLK15), transcript variant 4, mRNA. | | Down regulated | |
| MUC12 | PREDICTED: Homo sapiens mucin 12 (MUC12), mRNA. | | Down regulated | |
| PRAC | Homo sapiens small nuclear protein PRAC (PRAC), mRNA. | | Down regulated | |
| ANKRD38 | Homo sapiens ankyrin repeat domain 38 (ANKRD38), mRNA. | | Down regulated | |
| ITLN1 | Homo sapiens intelectin 1 (galactofuranose binding) (ITLN1), mRNA. | | Down regulated | |
| RPS23 | Homo sapiens ribosomal protein S23 (RPS23), mRNA. | | Down regulated | |
| HS.559682 | df01e07.w1 Morton Fetal Cochlea Homo sapiens cDNA clone IMAGE:2482260 3, mRNA sequence | | Down regulated | |
| GGT2 | Homo sapiens gamma-glutamyltransferase 2 (GGT2), mRNA. | | Down regulated | |
| TRPM6 | Homo sapiens transient receptor potential cation channel, subfamily M, member 6 (TRPM6), mRNA. | | Down regulated | |
| NEU4 | Homo sapiens sialidase 4 (NEU4), mRNA. | | Down regulated | |
| HOXA13 | Homo sapiens homeo box A13 (HOXA13), mRNA. | | Down regulated | |
| AKAP7 | Homo sapiens A kinase (PRKA) anchor protein 7 (AKAP7), transcript variant alpha, mRNA. | | Down regulated | |
| PNCK | Homo sapiens pregnancy upregulated non-ubiquitously expressed CaM kinase (PNCK), mRNA. | | Down regulated | |
| GALNT5 | Homo sapiens UDP-N-acetyl-alpha-D-galactosamine:polypeptide N-acetylgalactosaminyltransferase 5 (GalNAc-T5) (GALNT5), mRNA. | | Down regulated | |
| WNK2 | Homo sapiens WNK lysine deficient protein kinase 2 (WNK2), mRNA. | | Down regulated | |
| LOC401233 | Homo sapiens similar to HIV TAT specific factor 1; cofactor required for Tat activation of HIV-1 transcription (LOC401233), mRNA. | | Down regulated | |
| PCSK1N | Homo sapiens proprotein convertase subtilisin/kexin type 1 inhibitor (PCSK1N), mRNA. | | Down regulated | |
| PTPN21 | Homo sapiens protein tyrosine phosphatase, non-receptor type 21 (PTPN21), mRNA. | | Down regulated | |
| SLC45A3 | Homo sapiens solute carrier family 45, member 3 (SLC45A3), mRNA. | | Down regulated | |
| C12ORF34 | Homo sapiens chromosome 12 open reading frame 34 (C12orf34), mRNA. | | Down regulated | |
| GPR120 | Homo sapiens G protein-coupled receptor 120 (GPR120), mRNA. | | Down regulated | |
| KIAA0703 | Homo sapiens KIAA0703 gene product (KIAA0703), mRNA. | | Down regulated | |
| HS.249102 | xf03c11.x1 NCI_CGAP_Brn35 Homo sapiens cDNA clone IMAGE:2616980 3, mRNA sequence | | Down regulated | |
| LOC284422 | PREDICTED: Homo sapiens similar to HSPC323 (LOC284422), mRNA. | | Down regulated | |
| EXPH5 | Homo sapiens exophilin 5 (EXPH5), mRNA. | | Down regulated | |
| GHR | Homo sapiens growth hormone receptor (GHR), mRNA. | | Down regulated | |
| GNE | Homo sapiens glucosamine (UDP-N-acetyl)-2-epimerase/N-acetylmannosamine kinase (GNE), mRNA. | | Down regulated | |
| DKFZP564O0823 | Homo sapiens DKFZP564O0823 protein (DKFZP564O0823), mRNA. | | Down regulated | |
| SLC4A4 | Homo sapiens solute carrier family 4, sodium bicarbonate cotransporter, member 4 (SLC4A4), mRNA. | | Down regulated | |
| SCGB2A1 | Homo sapiens secretoglobin, family 2A, member 1 (SCGB2A1), mRNA. | | Down regulated | |
| FGFBP1 | Homo sapiens fibroblast growth factor binding protein 1 (FGFBP1), mRNA. | | Down regulated | |
| NKX2-2 | Homo sapiens NK2 homeobox 2 (NKX2-2), mRNA. | | Down regulated | |
| LOC642934 | PREDICTED: Homo sapiens hypothetical protein LOC642934 (LOC642934), mRNA. | | Down regulated | |
| CASD1 | Homo sapiens CAS1 domain containing 1 (CASD1), mRNA. | | Down regulated | |
| CCL28 | Homo sapiens chemokine (C-C motif) ligand 28 (CCL28), mRNA. | | Down regulated | |
| FAM101A | Homo sapiens family with sequence similarity 101, member A (FAM101A), mRNA. | | Down regulated | |
| FAM55A | Homo sapiens family with sequence similarity 55, member A (FAM55A), mRNA. | | Down regulated | |
| PON3 | Homo sapiens paraoxonase 3 (PON3), mRNA. | | Down regulated | |
| HHLA2 | Homo sapiens HERV-H LTR-associating 2 (HHLA2), mRNA. | | Down regulated | |
| SLC7A4 | Homo sapiens solute carrier family 7 (cationic amino acid transporter, y+ system), member 4 (SLC7A4), mRNA. | | Down regulated | |
| NEDD4L | Homo sapiens neural precursor cell expressed, developmentally down-regulated 4-like (NEDD4L), mRNA. | | Down regulated | |
| STARD10 | Homo sapiens START domain containing 10 (STARD10), mRNA. | | Down regulated | |
| QPRT | Homo sapiens quinolinate phosphoribosyltransferase (nicotinate-nucleotide pyrophosphorylase (carboxylating)) (QPRT), mRNA. | | Down regulated | |
| SCGN | Homo sapiens secretagogin, EF-hand calcium binding protein (SCGN), mRNA. | | Down regulated | |
| HS.126245 | CR743148 NCI_CGAP_GC4 Homo sapiens cDNA clone IMAGp971L0563 ; IMAGE:1550800 5, mRNA sequence | | Down regulated | |
| KIAA1715 | Homo sapiens KIAA1715 (KIAA1715), mRNA. | | Down regulated | |
| ATBF1 | Homo sapiens AT-binding transcription factor 1 (ATBF1), mRNA. | | Down regulated | |
| IL28RA | Homo sapiens interleukin 28 receptor, alpha (interferon, lambda receptor) (IL28RA), transcript variant 1, mRNA. | | Down regulated | |
| HS.556082 | Homo sapiens cDNA clone MGC:13035 IMAGE:3613409, complete cds | | Down regulated | |
| HS.547712 | Homo sapiens mRNA full length insert cDNA clone EUROIMAGE 1090207 | | Down regulated | |
| FLJ23152 | PREDICTED: Homo sapiens hypothetical gene supported by AK026805 (FLJ23152), mRNA. | | Down regulated | |
| CDCA7L | Homo sapiens cell division cycle associated 7-like (CDCA7L), mRNA. | | Down regulated | |
| TOM1L1 | Homo sapiens target of myb1 (chicken)-like 1 (TOM1L1), mRNA. | | Down regulated | |
| AGR2 | Homo sapiens anterior gradient homolog 2 (Xenopus laevis) (AGR2), mRNA. | | Down regulated | |
| CGNL1 | Homo sapiens cingulin-like 1 (CGNL1), mRNA. | | Down regulated | |
| ZDHHC23 | Homo sapiens zinc finger, DHHC-type containing 23 (ZDHHC23), mRNA. | | Down regulated | |
| ZNF91 | Homo sapiens zinc finger protein 91 (HPF7, HTF10) (ZNF91), mRNA. | | Down regulated | |
| NR3C2 | Homo sapiens nuclear receptor subfamily 3, group C, member 2 (NR3C2), mRNA. | | Down regulated | |
| ENTPD4 | Homo sapiens ectonucleoside triphosphate diphosphohydrolase 4 (ENTPD4), mRNA. | | Down regulated | |
| BARX2 | Homo sapiens BarH-like homeobox 2 (BARX2), mRNA. | | Down regulated | |
| FRMD3 | Homo sapiens FERM domain containing 3 (FRMD3), mRNA. | | Down regulated | |
| ACADSB | Homo sapiens acyl-Coenzyme A dehydrogenase, short/branched chain (ACADSB), nuclear gene encoding mitochondrial protein, mRNA. | | Down regulated | |
| TNRC9 | Homo sapiens TOX high mobility group box family member 3 (TOX3), mRNA. | | Down regulated | |
| FLJ20701 | Homo sapiens phosphotyrosine interaction domain containing 1 (PID1), mRNA. | | Down regulated | |
| C3ORF52 | Homo sapiens chromosome 3 open reading frame 52 (C3orf52), mRNA. | | Down regulated | |
| COBL | Homo sapiens cordon-bleu homolog (mouse) (COBL), mRNA. | | Down regulated | |
| PIN4 | Homo sapiens protein (peptidylprolyl cis/trans isomerase) NIMA-interacting, 4 (parvulin) (PIN4), mRNA. | | Down regulated | |
| PTPRO | Homo sapiens protein tyrosine phosphatase, receptor type, O (PTPRO), transcript variant 5, mRNA. | | Down regulated | |
| LGR4 | Homo sapiens leucine-rich repeat-containing G protein-coupled receptor 4 (LGR4), mRNA. | | Down regulated | |
| PRSS16 | Homo sapiens protease, serine, 16 (thymus) (PRSS16), mRNA. | | Down regulated | |
| PRELID2 | Homo sapiens PRELI domain containing 2 (PRELID2), transcript variant 2, mRNA. | | Down regulated | |
| ACF | Homo sapiens apobec-1 complementation factor (ACF), transcript variant 3, mRNA. | | Down regulated | |
| HS.159264 | Human clone 23948 mRNA sequence | | Down regulated | |
| KRT19 | Homo sapiens keratin 19 (KRT19), mRNA. | | Down regulated | |
| C6ORF85 | Homo sapiens chromosome 6 open reading frame 85 (C6orf85), mRNA. | | Down regulated | |
| ELOVL6 | Homo sapiens ELOVL family member 6, elongation of long chain fatty acids (FEN1/Elo2, SUR4/Elo3-like, yeast) (ELOVL6), mRNA. | | Down regulated | |
| C14ORF151 | Homo sapiens chromosome 14 open reading frame 151 (C14orf151), mRNA. | | Down regulated | |
| OXCT1 | Homo sapiens 3-oxoacid CoA transferase 1 (OXCT1), nuclear gene encoding mitochondrial protein, mRNA. | | Down regulated | |
| SMPD3 | Homo sapiens sphingomyelin phosphodiesterase 3, neutral membrane (neutral sphingomyelinase II) (SMPD3), mRNA. | | Down regulated | |
| IDH3A | Homo sapiens isocitrate dehydrogenase 3 (NAD+) alpha (IDH3A), nuclear gene encoding mitochondrial protein, mRNA. | | Down regulated | |
| SATB2 | Homo sapiens SATB homeobox 2 (SATB2), mRNA. | | Down regulated | |
| AHNAK | Homo sapiens AHNAK nucleoprotein (AHNAK), transcript variant 2, mRNA. | | Down regulated | |
| ACY1L2 | Homo sapiens aminoacylase 1-like 2 (ACY1L2), mRNA. | | Down regulated | |
| HS.282795 | Homo sapiens cDNA FLJ26512 fis, clone KDN07513 | | Down regulated | |
| PHLPP | Homo sapiens PH domain and leucine rich repeat protein phosphatase (PHLPP), mRNA. | | Down regulated | |
| IHH | Homo sapiens Indian hedgehog homolog (Drosophila) (IHH), mRNA. | | Down regulated | |
| ACTG2 | Homo sapiens actin, gamma 2, smooth muscle, enteric (ACTG2), mRNA. | | Down regulated | |
| MALL | Homo sapiens mal, T-cell differentiation protein-like (MALL), mRNA. | | Down regulated | |
| DNALI1 | Homo sapiens dynein, axonemal, light intermediate chain 1 (DNALI1), mRNA. | | Down regulated | |
| PLCE1 | Homo sapiens phospholipase C, epsilon 1 (PLCE1), mRNA. | | Down regulated | |
| HS.291319 | Homo sapiens mRNA; cDNA DKFZp779M2422 (from clone DKFZp779M2422) | | Down regulated | |
| ST6GALNAC1 | Homo sapiens ST6 (alpha-N-acetyl-neuraminyl-2,3-beta-galactosyl-1, 3)-N-acetylgalactosaminide alpha-2,6-sialyltransferase 1 (ST6GALNAC1), mRNA. | | Down regulated | |
| LOC647037 | PREDICTED: Homo sapiens similar to Peptidyl-prolyl cis-trans isomerase NIMA-interacting 4 (Rotamase Pin4) (PPIase Pin4) (Parvulin 14) (Par14) (Peptidyl-prolyl cis/trans isomerase EPVH) (hPar14) (LOC647037), mRNA. | | Down regulated | |
| PLEKHA6 | Homo sapiens pleckstrin homology domain containing, family A member 6 (PLEKHA6), mRNA. | | Down regulated | |
| ILDR1 | Homo sapiens immunoglobulin-like domain containing receptor 1 (ILDR1), mRNA. | | Down regulated | |
| CLCN2 | Homo sapiens chloride channel 2 (CLCN2), mRNA. | | Down regulated | |
| LOC389432 | Homo sapiens sterile alpha motif domain containing 5 (SAMD5), mRNA. | | Down regulated | |
| MICALCL | Homo sapiens MICAL C-terminal like (MICALCL), mRNA. | | Down regulated | |
| AP2B1 | Homo sapiens adaptor-related protein complex 2, beta 1 subunit (AP2B1), transcript variant 2, mRNA. | | Down regulated | |
| CFTR | Homo sapiens cystic fibrosis transmembrane conductance regulator, ATP-binding cassette (sub-family C, member 7) (CFTR), mRNA. | | Down regulated | |
| LOC653400 | PREDICTED: Homo sapiens similar to Occludin (LOC653400), mRNA. | | Down regulated | |
| MTM1 | Homo sapiens myotubularin 1 (MTM1), mRNA. | | Down regulated | |
| FLJ20152 | Homo sapiens hypothetical protein FLJ20152 (FLJ20152), transcript variant 1, mRNA. | | Down regulated | |
| CCNB2 | Homo sapiens cyclin B2 (CCNB2), mRNA. | | Down regulated | |
| HIGD1A | Homo sapiens HIG1 domain family, member 1A (HIGD1A), mRNA. | | Down regulated | |
| IGSF3 | Homo sapiens immunoglobulin superfamily, member 3 (IGSF3), transcript variant 1, mRNA. | | Down regulated | |
| LOC644743 | PREDICTED: Homo sapiens hypothetical protein LOC644743 (LOC644743), mRNA. | | Down regulated | |
| TESK2 | Homo sapiens testis-specific kinase 2 (TESK2), mRNA. | | Down regulated | |
| RICS | Homo sapiens Rho GTPase-activating protein (RICS), mRNA. | | Down regulated | |
| KIAA0672 | Homo sapiens KIAA0672 gene product (KIAA0672), mRNA. | | Down regulated | |
| S100A14 | Homo sapiens S100 calcium binding protein A14 (S100A14), mRNA. | | Down regulated | |
| EEF2K | Homo sapiens eukaryotic elongation factor-2 kinase (EEF2K), mRNA. | | Down regulated | |
| FAM3C | Homo sapiens family with sequence similarity 3, member C (FAM3C), transcript variant 2, mRNA. | | Down regulated | |
| POLE | Homo sapiens polymerase (DNA directed), epsilon (POLE), mRNA. | | Down regulated | |
| KIF22 | Homo sapiens kinesin family member 22 (KIF22), mRNA. | | Down regulated | |
| ZSWIM5 | PREDICTED: Homo sapiens zinc finger, SWIM-type containing 5 (ZSWIM5), mRNA. | | Down regulated | |
| ZNF702 | Homo sapiens zinc finger protein 702 (ZNF702), mRNA. | | Down regulated | |
| IRF6 | Homo sapiens interferon regulatory factor 6 (IRF6), mRNA. | | Down regulated | |
| MYO5C | Homo sapiens myosin VC (MYO5C), mRNA. | | Down regulated | |
| C14ORF147 | Homo sapiens chromosome 14 open reading frame 147 (C14orf147), mRNA. | | Down regulated | |
| ARL14 | Homo sapiens ADP-ribosylation factor-like 14 (ARL14), mRNA. | | Down regulated | |
| ANKS4B | Homo sapiens ankyrin repeat and sterile alpha motif domain containing 4B (ANKS4B), mRNA. | | Down regulated | |
| HS.19339 | Homo sapiens cDNA clone IMAGE:5263177 | | Down regulated | |
| PKP2 | Homo sapiens plakophilin 2 (PKP2), transcript variant 2a, mRNA. | | Down regulated | |
| KLF4 | Homo sapiens Kruppel-like factor 4 (gut) (KLF4), mRNA. | | Down regulated | |
| RBM38 | Homo sapiens RNA binding motif protein 38 (RBM38), transcript variant 2, mRNA. | | Down regulated | |
| KRT8 | Homo sapiens keratin 8 (KRT8), mRNA. | | Down regulated | |
| SPATA2 | Homo sapiens spermatogenesis associated 2 (SPATA2), mRNA. | | Down regulated | |
| LOC728758 | Homo sapiens hCG1789710 (LOC728758), mRNA. | | Down regulated | |
| UNC13B | Homo sapiens unc-13 homolog B (C. elegans) (UNC13B), mRNA. | | Down regulated | |
| C9ORF152 | Homo sapiens chromosome 9 open reading frame 152 (C9orf152), mRNA. | | Down regulated | |
| VIPR1 | Homo sapiens vasoactive intestinal peptide receptor 1 (VIPR1), mRNA. | | Down regulated | |
| GNAQ | Homo sapiens guanine nucleotide binding protein (G protein), q polypeptide (GNAQ), mRNA. | | Down regulated | |
| LOC387882 | Homo sapiens hypothetical protein (LOC387882), mRNA. | | Down regulated | |
| MED4 | Homo sapiens mediator complex subunit 4 (MED4), mRNA. | | Down regulated | |
| SEMA4G | Homo sapiens sema domain, immunoglobulin domain (Ig), transmembrane domain (TM) and short cytoplasmic domain, (semaphorin) 4G (SEMA4G), mRNA. | | Down regulated | |
| SCNN1A | Homo sapiens sodium channel, nonvoltage-gated 1 alpha (SCNN1A), mRNA. | | Down regulated | |
| MAL2 | Homo sapiens mal, T-cell differentiation protein 2 (MAL2), mRNA. | | Down regulated | |
| NANS | Homo sapiens N-acetylneuraminic acid synthase (sialic acid synthase) (NANS), mRNA. | | Down regulated | |
| SSPN | Homo sapiens sarcospan (Kras oncogene-associated gene) (SSPN), mRNA. | | Down regulated | |
| ZNF219 | Homo sapiens zinc finger protein 219 (ZNF219), mRNA. | | Down regulated | |
| RAVER2 | Homo sapiens ribonucleoprotein, PTB-binding 2 (RAVER2), mRNA. | | Down regulated | |
| KIAA1727 | Homo sapiens KIAA1727 protein (KIAA1727), mRNA. | | Down regulated | |
| TOP2A | Homo sapiens topoisomerase (DNA) II alpha 170kDa (TOP2A), mRNA. | | Down regulated | |
| SHRM | Homo sapiens shroom (SHRM), mRNA. | | Down regulated | |
| CDS1 | Homo sapiens CDP-diacylglycerol synthase (phosphatidate cytidylyltransferase) 1 (CDS1), mRNA. | | Down regulated | |
| GOLPH2 | Homo sapiens golgi phosphoprotein 2 (GOLPH2), transcript variant 2, mRNA. | | Down regulated | |
| CLDN23 | Homo sapiens claudin 23 (CLDN23), mRNA. | | Down regulated | |
| FAM36A | Homo sapiens family with sequence similarity 36, member A (FAM36A), mRNA. | | Down regulated | |
| FZD5 | Homo sapiens frizzled homolog 5 (Drosophila) (FZD5), mRNA. | | Down regulated | |
| C1ORF21 | Homo sapiens chromosome 1 open reading frame 21 (C1orf21), mRNA. | | Down regulated | |
| PEX1 | Homo sapiens peroxisome biogenesis factor 1 (PEX1), mRNA. | | Down regulated | |
| LRIG3 | Homo sapiens leucine-rich repeats and immunoglobulin-like domains 3 (LRIG3), mRNA. | | Down regulated | |
| TXNRD1 | Homo sapiens thioredoxin reductase 1 (TXNRD1), transcript variant 4, mRNA. | | Down regulated | |
| DAAM1 | Homo sapiens dishevelled associated activator of morphogenesis 1 (DAAM1), mRNA. | | Down regulated | |
| S100A16 | Homo sapiens S100 calcium binding protein A16 (S100A16), mRNA. | | Down regulated | |
| HS.276860 | 601441142F1 NIH_MGC_72 Homo sapiens cDNA clone IMAGE:3915971 5, mRNA sequence | | Down regulated | |
| CCDC47 | Homo sapiens coiled-coil domain containing 47 (CCDC47), mRNA. | | Down regulated | |
| EHF | Homo sapiens ets homologous factor (EHF), mRNA. | | Down regulated | |
| H1F0 | Homo sapiens H1 histone family, member 0 (H1F0), mRNA. | | Down regulated | |
| CXADR | Homo sapiens coxsackie virus and adenovirus receptor (CXADR), mRNA. | | Down regulated | |
| SLC22A5 | Homo sapiens solute carrier family 22 (organic cation transporter), member 5 (SLC22A5), mRNA. | | Down regulated | |
| ADCY6 | Homo sapiens adenylate cyclase 6 (ADCY6), transcript variant 1, mRNA. | | Down regulated | |
| PHB | Homo sapiens prohibitin (PHB), mRNA. | | Down regulated | |
| MGC52423 | Homo sapiens hypothetical protein MGC52423 (MGC52423), mRNA. | | Down regulated | |
| MTAC2D1 | Homo sapiens membrane targeting (tandem) C2 domain containing 1 (MTAC2D1), mRNA. | | Down regulated | |
| FLNB | Homo sapiens filamin B, beta (actin binding protein 278) (FLNB), mRNA. | | Down regulated | |
| XYLT2 | Homo sapiens xylosyltransferase II (XYLT2), mRNA. | | Down regulated | |
| C20ORF129 | Homo sapiens chromosome 20 open reading frame 129 (C20orf129), mRNA. | | Down regulated | |
| SLC35A3 | Homo sapiens solute carrier family 35 (UDP-N-acetylglucosamine (UDP-GlcNAc) transporter), member A3 (SLC35A3), mRNA. | | Down regulated | |
| TPMT | Homo sapiens thiopurine S-methyltransferase (TPMT), mRNA. | | Down regulated | |
| LOC653108 | PREDICTED: Homo sapiens similar to coxsackie virus and adenovirus receptor (LOC653108), mRNA. | | Down regulated | |
| EPS8L3 | Homo sapiens EPS8-like 3 (EPS8L3), transcript variant 3, mRNA. | | Down regulated | |
| FLJ20273 | Homo sapiens RNA-binding protein (FLJ20273), mRNA. | | Down regulated | |
| CAPN5 | Homo sapiens calpain 5 (CAPN5), mRNA. | | Down regulated | |
| SYTL4 | Homo sapiens synaptotagmin-like 4 (granuphilin-a) (SYTL4), mRNA. | | Down regulated | |
| ELLS1 | Homo sapiens hypothetical protein Ells1 (Ells1), mRNA. | | Down regulated | |
| HS.14555 | Homo sapiens cDNA: FLJ21513 fis, clone COL05778 | | Down regulated | |
| MEF2D | Homo sapiens myocyte enhancer factor 2D (MEF2D), mRNA. | | Down regulated | |
| GUCY2C | Homo sapiens guanylate cyclase 2C (heat stable enterotoxin receptor) (GUCY2C), mRNA. | | Down regulated | |
| GOLPH3L | Homo sapiens golgi phosphoprotein 3-like (GOLPH3L), mRNA. | | Down regulated | |
| PRC1 | Homo sapiens protein regulator of cytokinesis 1 (PRC1), transcript variant 1, mRNA. | | Down regulated | |
| MOBKL2B | Homo sapiens MOB1, Mps One Binder kinase activator-like 2B (yeast) (MOBKL2B), mRNA. | | Down regulated | |
| FLJ11017 | Homo sapiens chromosome 4 open reading frame 19 (C4orf19), mRNA. | | Down regulated | |
| MLR2 | Homo sapiens ligand-dependent corepressor (MLR2), mRNA. | | Down regulated | |
| FASN | Homo sapiens fatty acid synthase (FASN), mRNA. | | Down regulated | |
| C10ORF38 | Homo sapiens chromosome 10 open reading frame 38 (C10orf38), mRNA. | | Down regulated | |
| M160 | Homo sapiens scavenger receptor cysteine-rich type 1 protein M160 (M160), mRNA. | | Down regulated | |
| AURKA | Homo sapiens aurora kinase A (AURKA), transcript variant 3, mRNA. | | Down regulated | |
| DIP13B | Homo sapiens DIP13 beta (DIP13B), mRNA. | | Down regulated | |
| PYGB | Homo sapiens phosphorylase, glycogen; brain (PYGB), mRNA. | | Down regulated | |
| WASL | Homo sapiens Wiskott-Aldrich syndrome-like (WASL), mRNA. | | Down regulated | |
| IGF2BP2 | Homo sapiens insulin-like growth factor 2 mRNA binding protein 2 (IGF2BP2), transcript variant 1, mRNA. | | Down regulated | |
| CANT1 | Homo sapiens calcium activated nucleotidase 1 (CANT1), mRNA. | | Down regulated | |
| FTL | Homo sapiens ferritin, light polypeptide (FTL), mRNA. | | up regulated | |
| GNG10 | Homo sapiens guanine nucleotide binding protein (G protein), gamma 10 (GNG10), mRNA. | | up regulated | |
| DDIT3 | Homo sapiens DNA-damage-inducible transcript 3 (DDIT3), mRNA. | | up regulated | |
| CARD9 | Homo sapiens caspase recruitment domain family, member 9 (CARD9), mRNA. | | up regulated | |
| GYG1 | Homo sapiens glycogenin 1 (GYG1), mRNA. | | up regulated | |
| GLA | Homo sapiens galactosidase, alpha (GLA), mRNA. | | up regulated | |
| IGFBP4 | Homo sapiens insulin-like growth factor binding protein 4 (IGFBP4), mRNA. | | up regulated | |
| B3GALT6 | Homo sapiens UDP-Gal:betaGal beta 1,3-galactosyltransferase polypeptide 6 (B3GALT6), mRNA. | | up regulated | |
| FKBP11 | Homo sapiens FK506 binding protein 11, 19 kDa (FKBP11), mRNA. | | up regulated | |
| FKBP5 | Homo sapiens FK506 binding protein 5 (FKBP5), mRNA. | | up regulated | |
| ISG20L1 | Homo sapiens interferon stimulated exonuclease gene 20kDa-like 1 (ISG20L1), mRNA. | | up regulated | |
| NEU1 | Homo sapiens sialidase 1 (lysosomal sialidase) (NEU1), mRNA. | | up regulated | |
| DCBLD1 | Homo sapiens discoidin, CUB and LCCL domain containing 1 (DCBLD1), mRNA. | | up regulated | |
| PRCP | Homo sapiens prolylcarboxypeptidase (angiotensinase C) (PRCP), transcript variant 1, mRNA. | | up regulated | |
| OLFML3 | Homo sapiens olfactomedin-like 3 (OLFML3), mRNA. | | up regulated | |
| PDGFRA | Homo sapiens platelet-derived growth factor receptor, alpha polypeptide (PDGFRA), mRNA. | | up regulated | |
| TCN2 | Homo sapiens transcobalamin II; macrocytic anemia (TCN2), mRNA. | | up regulated | |
| TBC1D16 | Homo sapiens TBC1 domain family, member 16 (TBC1D16), mRNA. | | up regulated | |
| MFGE8 | Homo sapiens milk fat globule-EGF factor 8 protein (MFGE8), mRNA. | | up regulated | |
| ANXA5 | Homo sapiens annexin A5 (ANXA5), mRNA. | | up regulated | |
| LAMC1 | Homo sapiens laminin, gamma 1 (formerly LAMB2) (LAMC1), mRNA. | | up regulated | |
| GPT2 | Homo sapiens glutamic pyruvate transaminase (alanine aminotransferase) 2 (GPT2), mRNA. | | up regulated | |
| C20ORF98 | Homo sapiens neurensin 2 (NRSN2), mRNA. | | up regulated | |
| ARHGEF17 | Homo sapiens Rho guanine nucleotide exchange factor (GEF) 17 (ARHGEF17), mRNA. | | up regulated | |
| HEBP1 | Homo sapiens heme binding protein 1 (HEBP1), mRNA. | | up regulated | |
| A2M | Homo sapiens alpha-2-macroglobulin (A2M), mRNA. | | up regulated | |
| SPARC | Homo sapiens secreted protein, acidic, cysteine-rich (osteonectin) (SPARC), mRNA. | | up regulated | |
| C16ORF7 | Homo sapiens chromosome 16 open reading frame 7 (C16orf7), mRNA. | | up regulated | |
| TIMP1 | Homo sapiens TIMP metallopeptidase inhibitor 1 (TIMP1), mRNA. | | up regulated | |
| PCSK1 | Homo sapiens proprotein convertase subtilisin/kexin type 1 (PCSK1), mRNA. | | up regulated | |
| LOC388610 | Homo sapiens hypothetical LOC388610 (LOC388610), mRNA. | | up regulated | |
| OLFML1 | Homo sapiens olfactomedin-like 1 (OLFML1), mRNA. | | up regulated | |
| TSPAN9 | Homo sapiens tetraspanin 9 (TSPAN9), mRNA. | | up regulated | |
| D15WSU75E | Homo sapiens DNA segment, Chr 15, Wayne State University 75, expressed (D15Wsu75e), mRNA. | | up regulated | |
| NUAK1 | Homo sapiens NUAK family, SNF1-like kinase, 1 (NUAK1), mRNA. | | up regulated | |
| GJC1 | Homo sapiens gap junction protein, chi 1, 31.9kDa (GJC1), mRNA. | | up regulated | |
| PPM1F | Homo sapiens protein phosphatase 1F (PP2C domain containing) (PPM1F), mRNA. | | up regulated | |
| LOC642113 | PREDICTED: Homo sapiens similar to Ig kappa chain V-III region HAH precursor (LOC642113), mRNA. | | up regulated | |
| PODN | Homo sapiens podocan (PODN), mRNA. | | up regulated | |
| SNTB1 | Homo sapiens syntrophin, beta 1 (dystrophin-associated protein A1, 59kDa, basic component 1) (SNTB1), mRNA. | | up regulated | |
| SLC5A8 | Homo sapiens solute carrier family 5 (iodide transporter), member 8 (SLC5A8), mRNA. | | up regulated | |
| PCDH18 | Homo sapiens protocadherin 18 (PCDH18), mRNA. | | up regulated | |
| CTSB | Homo sapiens cathepsin B (CTSB), transcript variant 1, mRNA. | | up regulated | |
| RGS5 | Homo sapiens regulator of G-protein signalling 5 (RGS5), mRNA. | | up regulated | |
| TCF21 | Homo sapiens transcription factor 21 (TCF21), transcript variant 2, mRNA. | | up regulated | |
| IL32 | Homo sapiens interleukin 32 (IL32), transcript variant 3, mRNA. | | up regulated | |
| HS.505676 | Homo sapiens cDNA FLJ33772 fis, clone BRSSN2000175 | | up regulated | |
| ADFP | Homo sapiens adipose differentiation-related protein (ADFP), mRNA. | | up regulated | |
| SLC27A1 | Homo sapiens solute carrier family 27 (fatty acid transporter), member 1 (SLC27A1), mRNA. | | up regulated | |
| PTGER2 | Homo sapiens prostaglandin E receptor 2 (subtype EP2), 53kDa (PTGER2), mRNA. | | up regulated | |
| LMCD1 | Homo sapiens LIM and cysteine-rich domains 1 (LMCD1), mRNA. | | up regulated | |
| NR1H3 | Homo sapiens nuclear receptor subfamily 1, group H, member 3 (NR1H3), mRNA. | | up regulated | |
| WDR27 | Homo sapiens WD repeat domain 27 (WDR27), mRNA. | | up regulated | |
| ESAM | Homo sapiens endothelial cell adhesion molecule (ESAM), mRNA. | | up regulated | |
| LDB2 | Homo sapiens LIM domain binding 2 (LDB2), mRNA. | | up regulated | |
| HS.481464 | Homo sapiens cDNA FLJ13116 fis, clone NT2RP3002659 | | up regulated | |
| STAB1 | Homo sapiens stabilin 1 (STAB1), mRNA. | | up regulated | |
| LEPRE1 | Homo sapiens leucine proline-enriched proteoglycan (leprecan) 1 (LEPRE1), mRNA. | | up regulated | |
| AXL | Homo sapiens AXL receptor tyrosine kinase (AXL), transcript variant 1, mRNA. | | up regulated | |
| TAP1 | Homo sapiens transporter 1, ATP-binding cassette, sub-family B (MDR/TAP) (TAP1), mRNA. | | up regulated | |
| HBEGF | Homo sapiens heparin-binding EGF-like growth factor (HBEGF), mRNA. | | up regulated | |
| LIPA | Homo sapiens lipase A, lysosomal acid, cholesterol esterase (Wolman disease) (LIPA), mRNA. | | up regulated | |
| MPDZ | Homo sapiens multiple PDZ domain protein (MPDZ), mRNA. | | up regulated | |
| APH1B | Homo sapiens anterior pharynx defective 1 homolog B (C. elegans) (APH1B), mRNA. | | up regulated | |
| IDUA | Homo sapiens iduronidase, alpha-L- (IDUA), mRNA. | | up regulated | |
| NOD27 | Homo sapiens nucleotide-binding oligomerization domains 27 (NOD27), mRNA. | | up regulated | |
| C20ORF46 | Homo sapiens chromosome 20 open reading frame 46 (C20orf46), mRNA. | | up regulated | |
| PLVAP | Homo sapiens plasmalemma vesicle associated protein (PLVAP), mRNA. | | up regulated | |
| GPX3 | Homo sapiens glutathione peroxidase 3 (plasma) (GPX3), mRNA. | | up regulated | |
| HSD3B7 | Homo sapiens hydroxy-delta-5-steroid dehydrogenase, 3 beta- and steroid delta-isomerase 7 (HSD3B7), mRNA. | | up regulated | |
| PRKCDBP | Homo sapiens protein kinase C, delta binding protein (PRKCDBP), mRNA. | | up regulated | |
| FLJ11000 | Homo sapiens hypothetical protein FLJ11000 (FLJ11000), mRNA. | | up regulated | |
| SELM | Homo sapiens selenoprotein M (SELM), mRNA. | | up regulated | |
| LAMA4 | Homo sapiens laminin, alpha 4 (LAMA4), mRNA. | | up regulated | |
| EFEMP2 | Homo sapiens EGF-containing fibulin-like extracellular matrix protein 2 (EFEMP2), mRNA. | | up regulated | |
| LOC388588 | PREDICTED: Homo sapiens hypothetical LOC388588, transcript variant 3 (LOC388588), mRNA. | | up regulated | |
| TICAM2 | Homo sapiens toll-like receptor adaptor molecule 2 (TICAM2), mRNA. | | up regulated | |
| CYBRD1 | Homo sapiens cytochrome b reductase 1 (CYBRD1), mRNA. | | up regulated | |
| PTHR1 | Homo sapiens parathyroid hormone receptor 1 (PTHR1), mRNA. | | up regulated | |
| LAMA2 | Homo sapiens laminin, alpha 2 (merosin, congenital muscular dystrophy) (LAMA2), transcript variant 1, mRNA. | | up regulated | |
| CD74 | Homo sapiens CD74 molecule, major histocompatibility complex, class II invariant chain (CD74), transcript variant 2, mRNA. | | up regulated | |
| AGTRL1 | Homo sapiens angiotensin II receptor-like 1 (AGTRL1), mRNA. | | up regulated | |
| ADCY4 | Homo sapiens adenylate cyclase 4 (ADCY4), mRNA. | | up regulated | |
| HEYL | Homo sapiens hairy/enhancer-of-split related with YRPW motif-like (HEYL), mRNA. | | up regulated | |
| SERPINH1 | Homo sapiens serpin peptidase inhibitor, clade H (heat shock protein 47), member 1, (collagen binding protein 1) (SERPINH1), mRNA. | | up regulated | |
| SCNN1D | Homo sapiens sodium channel, nonvoltage-gated 1, delta (SCNN1D), mRNA. | | up regulated | |
| AP1S2 | Homo sapiens adaptor-related protein complex 1, sigma 2 subunit (AP1S2), mRNA. | | up regulated | |
| AOAH | Homo sapiens acyloxyacyl hydrolase (neutrophil) (AOAH), mRNA. | | up regulated | |
| HS.548415 | wv06f12.x1 NCI_CGAP_Kid3 Homo sapiens cDNA clone IMAGE:2528783 3 similar to gb:X17115 IG MU CHAIN C REGION (HUMAN);, mRNA sequence | | up regulated | |
| BTN3A3 | Homo sapiens butyrophilin, subfamily 3, member A3 (BTN3A3), transcript variant 1, mRNA. | | up regulated | |
| COL14A1 | Homo sapiens collagen, type XIV, alpha 1 (undulin) (COL14A1), mRNA. | | up regulated | |
| CENTA2 | Homo sapiens centaurin, alpha 2 (CENTA2), mRNA. | | up regulated | |
| TIE1 | Homo sapiens tyrosine kinase with immunoglobulin-like and EGF-like domains 1 (TIE1), mRNA. | | up regulated | |
| TNMD | Homo sapiens tenomodulin (TNMD), mRNA. | | up regulated | |
| GJA1 | Homo sapiens gap junction protein, alpha 1, 43kDa (connexin 43) (GJA1), mRNA. | | up regulated | |
| SPARCL1 | Homo sapiens SPARC-like 1 (mast9, hevin) (SPARCL1), mRNA. | | up regulated | |
| PROCR | Homo sapiens protein C receptor, endothelial (EPCR) (PROCR), mRNA. | | up regulated | |
| ADAMDEC1 | Homo sapiens ADAM-like, decysin 1 (ADAMDEC1), mRNA. | | up regulated | |
| GSPT2 | Homo sapiens G1 to S phase transition 2 (GSPT2), mRNA. | | up regulated | |
| C6ORF189 | PREDICTED: Homo sapiens chromosome 6 open reading frame 189 (C6orf189), mRNA. | | up regulated | |
| LOX | Homo sapiens lysyl oxidase (LOX), mRNA. | | up regulated | |
| C1ORF78 | Homo sapiens chromosome 1 open reading frame 78 (C1orf78), mRNA. | | up regulated | |
| HRASLS3 | Homo sapiens HRAS-like suppressor 3 (HRASLS3), mRNA. | | up regulated | |
| NETO2 | Homo sapiens neuropilin (NRP) and tolloid (TLL)-like 2 (NETO2), mRNA. | | up regulated | |
| BNIP3 | Homo sapiens BCL2/adenovirus E1B 19kDa interacting protein 3 (BNIP3), nuclear gene encoding mitochondrial protein, mRNA. | | up regulated | |
| AKR1B1 | Homo sapiens aldo-keto reductase family 1, member B1 (aldose reductase) (AKR1B1), mRNA. | | up regulated | |
| LGALS1 | Homo sapiens lectin, galactoside-binding, soluble, 1 (galectin 1) (LGALS1), mRNA. | | up regulated | |
| PLXDC2 | Homo sapiens plexin domain containing 2 (PLXDC2), mRNA. | | up regulated | |
| ZNF467 | Homo sapiens zinc finger protein 467 (ZNF467), mRNA. | | up regulated | |
| C10ORF10 | Homo sapiens chromosome 10 open reading frame 10 (C10orf10), mRNA. | | up regulated | |
| GBGT1 | Homo sapiens globoside alpha-1,3-N-acetylgalactosaminyltransferase 1 (GBGT1), mRNA. | | up regulated | |
| LOC441019 | PREDICTED: Homo sapiens hypothetical LOC441019 (LOC441019), mRNA. | | up regulated | |
| LOC653371 | PREDICTED: Homo sapiens similar to Baculoviral IAP repeat-containing protein 1 (Neuronal apoptosis inhibitory protein) (LOC653371), mRNA. | | up regulated | |
| MGC24103 | PREDICTED: Homo sapiens hypothetical protein MGC24103 (MGC24103), misc RNA. | | up regulated | |
| RPESP | Homo sapiens RPE-spondin (RPESP), mRNA. | | up regulated | |
| KCNJ2 | Homo sapiens potassium inwardly-rectifying channel, subfamily J, member 2 (KCNJ2), mRNA. | | up regulated | |
| TARSL2 | Homo sapiens threonyl-tRNA synthetase-like 2 (TARSL2), mRNA. | | up regulated | |
| TRIB3 | Homo sapiens tribbles homolog 3 (Drosophila) (TRIB3), mRNA. | | up regulated | |
| TNFRSF12A | Homo sapiens tumor necrosis factor receptor superfamily, member 12A (TNFRSF12A), mRNA. | | up regulated | |
| RARRES3 | Homo sapiens retinoic acid receptor responder (tazarotene induced) 3 (RARRES3), mRNA. | | up regulated | |
| GLT8D2 | Homo sapiens glycosyltransferase 8 domain containing 2 (GLT8D2), mRNA. | | up regulated | |
| PSMB9 | Homo sapiens proteasome (prosome, macropain) subunit, beta type, 9 (large multifunctional peptidase 2) (PSMB9), transcript variant 1, mRNA. | | up regulated | |
| NPL | Homo sapiens N-acetylneuraminate pyruvate lyase (dihydrodipicolinate synthase) (NPL), mRNA. | | up regulated | |
| KCNS3 | Homo sapiens potassium voltage-gated channel, delayed-rectifier, subfamily S, member 3 (KCNS3), mRNA. | | up regulated | |
| ENG | Homo sapiens endoglin (Osler-Rendu-Weber syndrome 1) (ENG), mRNA. | | up regulated | |
| CTSC | Homo sapiens cathepsin C (CTSC), transcript variant 1, mRNA. | | up regulated | |
| C1S | Homo sapiens complement component 1, s subcomponent (C1S), transcript variant 1, mRNA. | | up regulated | |
| EMILIN1 | Homo sapiens elastin microfibril interfacer 1 (EMILIN1), mRNA. | | up regulated | |
| GIMAP5 | Homo sapiens GTPase, IMAP family member 5 (GIMAP5), mRNA. | | up regulated | |
| ABLIM3 | Homo sapiens actin binding LIM protein family, member 3 (ABLIM3), mRNA. | | up regulated | |
| GLIPR1 | Homo sapiens GLI pathogenesis-related 1 (glioma) (GLIPR1), mRNA. | | up regulated | |
| COL4A2 | Homo sapiens collagen, type IV, alpha 2 (COL4A2), mRNA. | | up regulated | |
| REM1 | Homo sapiens RAS (RAD and GEM)-like GTP-binding 1 (REM1), mRNA. | | up regulated | |
| PECAM1 | Homo sapiens platelet/endothelial cell adhesion molecule (CD31 antigen) (PECAM1), mRNA. | | up regulated | |
| GABRP | Homo sapiens gamma-aminobutyric acid (GABA) A receptor, pi (GABRP), mRNA. | | up regulated | |
| C1QTNF5 | Homo sapiens C1q and tumor necrosis factor related protein 5 (C1QTNF5), mRNA. | | up regulated | |
| LMTK3 | PREDICTED: Homo sapiens lemur tyrosine kinase 3 (LMTK3), mRNA. | | up regulated | |
| CFB | Homo sapiens complement factor B (CFB), mRNA. | | up regulated | |
| TGFBI | Homo sapiens transforming growth factor, beta-induced, 68kDa (TGFBI), mRNA. | | up regulated | |
| IGFBP7 | Homo sapiens insulin-like growth factor binding protein 7 (IGFBP7), mRNA. | | up regulated | |
| ECM1 | Homo sapiens extracellular matrix protein 1 (ECM1), transcript variant 2, mRNA. | | up regulated | |
| AMICA1 | Homo sapiens adhesion molecule, interacts with CXADR antigen 1 (AMICA1), mRNA. | | up regulated | |
| LHFP | Homo sapiens lipoma HMGIC fusion partner (LHFP), mRNA. | | up regulated | |
| HLA-DMA | Homo sapiens major histocompatibility complex, class II, DM alpha (HLA-DMA), mRNA. | | up regulated | |
| PPM1M | Homo sapiens protein phosphatase 1M (PP2C domain containing) (PPM1M), mRNA. | | up regulated | |
| ROBO3 | Homo sapiens roundabout, axon guidance receptor, homolog 3 (Drosophila) (ROBO3), mRNA. | | up regulated | |
| FLJ39378 | Homo sapiens hypothetical protein FLJ39378 (FLJ39378), mRNA. | | up regulated | |
| GIMAP4 | Homo sapiens GTPase, IMAP family member 4 (GIMAP4), mRNA. | | up regulated | |
| RBP1 | Homo sapiens retinol binding protein 1, cellular (RBP1), mRNA. | | up regulated | |
| TM4SF18 | Homo sapiens transmembrane 4 L six family member 18 (TM4SF18), mRNA. | | up regulated | |
| C2ORF11 | Homo sapiens raftlin family member 2 (RFTN2), mRNA. | | up regulated | |
| CX3CL1 | Homo sapiens chemokine (C-X3-C motif) ligand 1 (CX3CL1), mRNA. | | up regulated | |
| HS.535044 | PREDICTED: Homo sapiens similar to FLJ00290 protein (LOC441310), mRNA | | up regulated | |
| TPST1 | Homo sapiens tyrosylprotein sulfotransferase 1 (TPST1), mRNA. | | up regulated | |
| CPA3 | Homo sapiens carboxypeptidase A3 (mast cell) (CPA3), mRNA. | | up regulated | |
| IL3RA | Homo sapiens interleukin 3 receptor, alpha (low affinity) (IL3RA), mRNA. | | up regulated | |
| DCN | Homo sapiens decorin (DCN), transcript variant C, mRNA. | | up regulated | |
| BCL6B | Homo sapiens B-cell CLL/lymphoma 6, member B (zinc finger protein) (BCL6B), mRNA. | | up regulated | |
| HTRA1 | Homo sapiens HtrA serine peptidase 1 (HTRA1), mRNA. | | up regulated | |
| PTGER1 | Homo sapiens prostaglandin E receptor 1 (subtype EP1), 42kDa (PTGER1), mRNA. | | up regulated | |
| CDH11 | Homo sapiens cadherin 11, type 2, OB-cadherin (osteoblast) (CDH11), mRNA. | | up regulated | |
| BST2 | Homo sapiens bone marrow stromal cell antigen 2 (BST2), mRNA. | | up regulated | |
| STOM | Homo sapiens stomatin (STOM), transcript variant 1, mRNA. | | up regulated | |
| EPSTI1 | Homo sapiens epithelial stromal interaction 1 (breast) (EPSTI1), transcript variant 2, mRNA. | | up regulated | |
| CLEC11A | Homo sapiens C-type lectin domain family 11, member A (CLEC11A), mRNA. | | up regulated | |
| GBP2 | Homo sapiens guanylate binding protein 2, interferon-inducible (GBP2), mRNA. | | up regulated | |
| LRRC32 | Homo sapiens leucine rich repeat containing 32 (LRRC32), mRNA. | | up regulated | |
| PCCA | Homo sapiens propionyl Coenzyme A carboxylase, alpha polypeptide (PCCA), mRNA. | | up regulated | |
| APOC1 | Homo sapiens apolipoprotein C-I (APOC1), mRNA. | | up regulated | |
| HEY2 | Homo sapiens hairy/enhancer-of-split related with YRPW motif 2 (HEY2), mRNA. | | up regulated | |
| HLA-DQA1 | PREDICTED: Homo sapiens major histocompatibility complex, class II, DQ alpha 1, transcript variant 2 (HLA-DQA1), mRNA. | | up regulated | |
| NNMT | Homo sapiens nicotinamide N-methyltransferase (NNMT), mRNA. | | up regulated | |
| DDIT4 | Homo sapiens DNA-damage-inducible transcript 4 (DDIT4), mRNA. | | up regulated | |
| LOXL3 | Homo sapiens lysyl oxidase-like 3 (LOXL3), mRNA. | | up regulated | |
| CBR3 | Homo sapiens carbonyl reductase 3 (CBR3), mRNA. | | up regulated | |
| HLA-DPA1 | Homo sapiens major histocompatibility complex, class II, DP alpha 1 (HLA-DPA1), mRNA. | | up regulated | |
| C16ORF30 | Homo sapiens chromosome 16 open reading frame 30 (C16orf30), mRNA. | | up regulated | |
| DMBT1 | Homo sapiens deleted in malignant brain tumors 1 (DMBT1), transcript variant 3, mRNA. | | up regulated | |
| HLA-DPB1 | Homo sapiens major histocompatibility complex, class II, DP beta 1 (HLA-DPB1), mRNA. | | up regulated | |
| SCPEP1 | Homo sapiens serine carboxypeptidase 1 (SCPEP1), mRNA. | | up regulated | |
| TSPAN4 | Homo sapiens tetraspanin 4 (TSPAN4), transcript variant 2, mRNA. | | up regulated | |
| CRISPLD2 | Homo sapiens cysteine-rich secretory protein LCCL domain containing 2 (CRISPLD2), mRNA. | | up regulated | |
| GPR116 | Homo sapiens G protein-coupled receptor 116 (GPR116), mRNA. | | up regulated | |
| HS.132272 | Homo sapiens mRNA; cDNA DKFZp762D186 (from clone DKFZp762D186) | | up regulated | |
| SMOX | Homo sapiens spermine oxidase (SMOX), transcript variant 4, mRNA. | | up regulated | |
| GPBAR1 | Homo sapiens G protein-coupled bile acid receptor 1 (GPBAR1), transcript variant 1, mRNA. | | up regulated | |
| CSF1R | Homo sapiens colony stimulating factor 1 receptor, formerly McDonough feline sarcoma viral (v-fms) oncogene homolog (CSF1R), mRNA. | | up regulated | |
| RENBP | Homo sapiens renin binding protein (RENBP), mRNA. | | up regulated | |
| CLEC14A | Homo sapiens C-type lectin domain family 14, member A (CLEC14A), mRNA. | | up regulated | |
| CDH13 | Homo sapiens cadherin 13, H-cadherin (heart) (CDH13), mRNA. | | up regulated | |
| VIP | Homo sapiens vasoactive intestinal peptide (VIP), transcript variant 1, mRNA. | | up regulated | |
| BLVRA | Homo sapiens biliverdin reductase A (BLVRA), mRNA. | | up regulated | |
| RASIP1 | Homo sapiens Ras interacting protein 1 (RASIP1), mRNA. | | up regulated | |
| LAP3 | Homo sapiens leucine aminopeptidase 3 (LAP3), mRNA. | | up regulated | |
| PTGDS | Homo sapiens prostaglandin D2 synthase 21kDa (brain) (PTGDS), mRNA. | | up regulated | |
| FES | Homo sapiens feline sarcoma oncogene (FES), mRNA. | | up regulated | |
| ZNF683 | Homo sapiens zinc finger protein 683 (ZNF683), mRNA. | | up regulated | |
| PER3 | Homo sapiens period homolog 3 (Drosophila) (PER3), mRNA. | | up regulated | |
| MS4A6A | Homo sapiens membrane-spanning 4-domains, subfamily A, member 6A (MS4A6A), transcript variant 2, mRNA. | | up regulated | |
| MS4A8B | Homo sapiens membrane-spanning 4-domains, subfamily A, member 8B (MS4A8B), mRNA. | | up regulated | |
| COL16A1 | Homo sapiens collagen, type XVI, alpha 1 (COL16A1), mRNA. | | up regulated | |
| RASL11B | Homo sapiens RAS-like, family 11, member B (RASL11B), mRNA. | | up regulated | |
| CDH5 | Homo sapiens cadherin 5, type 2, VE-cadherin (vascular epithelium) (CDH5), mRNA. | | up regulated | |
| ASB16 | Homo sapiens ankyrin repeat and SOCS box-containing 16 (ASB16), mRNA. | | up regulated | |
| STARD8 | Homo sapiens START domain containing 8 (STARD8), mRNA. | | up regulated | |
| LY96 | Homo sapiens lymphocyte antigen 96 (LY96), mRNA. | | up regulated | |
| UBTD1 | Homo sapiens ubiquitin domain containing 1 (UBTD1), mRNA. | | up regulated | |
| ANGPT1 | Homo sapiens angiopoietin 1 (ANGPT1), mRNA. | | up regulated | |
| PLA2G3 | Homo sapiens phospholipase A2, group III (PLA2G3), mRNA. | | up regulated | |
| CD248 | Homo sapiens CD248 molecule, endosialin (CD248), mRNA. | | up regulated | |
| CLEC1A | Homo sapiens C-type lectin domain family 1, member A (CLEC1A), mRNA. | | up regulated | |
| C1ORF54 | Homo sapiens chromosome 1 open reading frame 54 (C1orf54), mRNA. | | up regulated | |
| ITGB2 | Homo sapiens integrin, beta 2 (antigen CD18 (p95), lymphocyte function-associated antigen 1; macrophage antigen 1 (mac-1) beta subunit) (ITGB2), mRNA. | | up regulated | |
| INHBB | Homo sapiens inhibin, beta B (activin AB beta polypeptide) (INHBB), mRNA. | | up regulated | |
| NOTCH3 | Homo sapiens Notch homolog 3 (Drosophila) (NOTCH3), mRNA. | | up regulated | |
| RCN3 | Homo sapiens reticulocalbin 3, EF-hand calcium binding domain (RCN3), mRNA. | | up regulated | |
| TPSAB1 | Homo sapiens tryptase alpha/beta 1 (TPSAB1), mRNA. | | up regulated | |
| CD300LF | Homo sapiens CD300 molecule-like family member f (CD300LF), mRNA. | | up regulated | |
| PCDH17 | Homo sapiens protocadherin 17 (PCDH17), mRNA. | | up regulated | |
| SOX18 | Homo sapiens SRY (sex determining region Y)-box 18 (SOX18), mRNA. | | up regulated | |
| SNAI2 | Homo sapiens snail homolog 2 (Drosophila) (SNAI2), mRNA. | | up regulated | |
| RGS18 | Homo sapiens regulator of G-protein signalling 18 (RGS18), mRNA. | | up regulated | |
| MXRA5 | Homo sapiens matrix-remodelling associated 5 (MXRA5), mRNA. | | up regulated | |
| APOE | Homo sapiens apolipoprotein E (APOE), mRNA. | | up regulated | |
| AGT | Homo sapiens angiotensinogen (serpin peptidase inhibitor, clade A, member 8) (AGT), mRNA. | | up regulated | |
| DLG4 | Homo sapiens discs, large homolog 4 (Drosophila) (DLG4), mRNA. | | up regulated | |
| RAB31 | Homo sapiens RAB31, member RAS oncogene family (RAB31), mRNA. | | up regulated | |
| C1QC | Homo sapiens complement component 1, q subcomponent, C chain (C1QC), mRNA. | | up regulated | |
| MGC17839 | Homo sapiens transmembrane protein 136 (TMEM136), mRNA. | | up regulated | |
| CPXM2 | Homo sapiens carboxypeptidase X (M14 family), member 2 (CPXM2), mRNA. | | up regulated | |
| THY1 | Homo sapiens Thy-1 cell surface antigen (THY1), mRNA. | | up regulated | |
| NINJ1 | Homo sapiens ninjurin 1 (NINJ1), mRNA. | | up regulated | |
| ZMYND15 | Homo sapiens zinc finger, MYND-type containing 15 (ZMYND15), mRNA. | | up regulated | |
| EDNRA | Homo sapiens endothelin receptor type A (EDNRA), mRNA. | | up regulated | |
| RIS1 | Homo sapiens Ras-induced senescence 1 (RIS1), mRNA. | | up regulated | |
| CD55 | Homo sapiens CD55 molecule, decay accelerating factor for complement (Cromer blood group) (CD55), mRNA. | | up regulated | |
| TMEM91 | Homo sapiens transmembrane protein 91 (TMEM91), mRNA. | | up regulated | |
| COL6A3 | Homo sapiens collagen, type VI, alpha 3 (COL6A3), transcript variant 2, mRNA. | | up regulated | |
| SERPINA1 | Homo sapiens serpin peptidase inhibitor, clade A (alpha-1 antiproteinase, antitrypsin), member 1 (SERPINA1), transcript variant 1, mRNA. | | up regulated | |
| CSPG2 | Homo sapiens versican (VCAN), mRNA. | | up regulated | |
| PHLDA3 | Homo sapiens pleckstrin homology-like domain, family A, member 3 (PHLDA3), mRNA. | | up regulated | |
| COL15A1 | Homo sapiens collagen, type XV, alpha 1 (COL15A1), mRNA. | | up regulated | |
| SUSD2 | Homo sapiens sushi domain containing 2 (SUSD2), mRNA. | | up regulated | |
| GNG11 | Homo sapiens guanine nucleotide binding protein (G protein), gamma 11 (GNG11), mRNA. | | up regulated | |
| LST1 | Homo sapiens leukocyte specific transcript 1 (LST1), transcript variant 5, mRNA. | | up regulated | |
| LOC647134 | PREDICTED: Homo sapiens similar to Ig heavy chain V-III region VH26 precursor (LOC647134), mRNA. | | up regulated | |
| PAPPA | Homo sapiens pregnancy-associated plasma protein A, pappalysin 1 (PAPPA), mRNA. | | up regulated | |
| MMP2 | Homo sapiens matrix metallopeptidase 2 (gelatinase A, 72kDa gelatinase, 72kDa type IV collagenase) (MMP2), mRNA. | | up regulated | |
| SPAG4 | Homo sapiens sperm associated antigen 4 (SPAG4), mRNA. | | up regulated | |
| C10ORF81 | Homo sapiens chromosome 10 open reading frame 81 (C10orf81), mRNA. | | up regulated | |
| MS4A7 | Homo sapiens membrane-spanning 4-domains, subfamily A, member 7 (MS4A7), transcript variant 2, mRNA. | | up regulated | |
| SLITL2 | Homo sapiens slit-like 2 (Drosophila) (SLITL2), mRNA. | | up regulated | |
| MESP1 | Homo sapiens mesoderm posterior 1 homolog (mouse) (MESP1), mRNA. | | up regulated | |
| ECAT11 | Homo sapiens LINE-1 type transposase domain containing 1 (L1TD1), mRNA. | | up regulated | |
| TNFSF7 | Homo sapiens tumor necrosis factor (ligand) superfamily, member 7 (TNFSF7), mRNA. | | up regulated | |
| IFI30 | Homo sapiens interferon, gamma-inducible protein 30 (IFI30), mRNA. | | up regulated | |
| VAMP5 | Homo sapiens vesicle-associated membrane protein 5 (myobrevin) (VAMP5), mRNA. | | up regulated | |
| LRRC33 | Homo sapiens leucine rich repeat containing 33 (LRRC33), mRNA. | | up regulated | |
| IL18R1 | Homo sapiens interleukin 18 receptor 1 (IL18R1), mRNA. | | up regulated | |
| KCNJ8 | Homo sapiens potassium inwardly-rectifying channel, subfamily J, member 8 (KCNJ8), mRNA. | | up regulated | |
| COL23A1 | Homo sapiens collagen, type XXIII, alpha 1 (COL23A1), mRNA. | | up regulated | |
| ATHL1 | Homo sapiens ATH1, acid trehalase-like 1 (yeast) (ATHL1), mRNA. | | up regulated | |
| SVEP1 | Homo sapiens sushi, von Willebrand factor type A, EGF and pentraxin domain containing 1 (SVEP1), mRNA. | | up regulated | |
| AIF1 | Homo sapiens allograft inflammatory factor 1 (AIF1), transcript variant 2, mRNA. | | up regulated | |
| NOTCH4 | Homo sapiens Notch homolog 4 (Drosophila) (NOTCH4), mRNA. | | up regulated | |
| TLR4 | Homo sapiens toll-like receptor 4 (TLR4), transcript variant 4, mRNA. | | up regulated | |
| MSX1 | Homo sapiens msh homeo box homolog 1 (Drosophila) (MSX1), mRNA. | | up regulated | |
| CEECAM1 | Homo sapiens cerebral endothelial cell adhesion molecule 1 (CEECAM1), mRNA. | | up regulated | |
| KLRF1 | Homo sapiens killer cell lectin-like receptor subfamily F, member 1 (KLRF1), mRNA. | | up regulated | |
| SNCAIP | Homo sapiens synuclein, alpha interacting protein (synphilin) (SNCAIP), mRNA. | | up regulated | |
| COL18A1 | Homo sapiens collagen, type XVIII, alpha 1 (COL18A1), transcript variant 2, mRNA. | | up regulated | |
| TYROBP | Homo sapiens TYRO protein tyrosine kinase binding protein (TYROBP), transcript variant 1, mRNA. | | up regulated | |
| SOD2 | Homo sapiens superoxide dismutase 2, mitochondrial (SOD2), nuclear gene encoding mitochondrial protein, transcript variant 1, mRNA. | | up regulated | |
| ANGPT2 | Homo sapiens angiopoietin 2 (ANGPT2), mRNA. | | up regulated | |
| EBF3 | Homo sapiens early B-cell factor 3 (EBF3), mRNA. | | up regulated | |
| LOC647108 | PREDICTED: Homo sapiens hypothetical protein LOC647108 (LOC647108), mRNA. | | up regulated | |
| ARP10 | Homo sapiens apolipoprotein B mRNA editing enzyme, catalytic polypeptide-like 3H (APOBEC3H), mRNA. | | up regulated | |
| FZD9 | Homo sapiens frizzled homolog 9 (Drosophila) (FZD9), mRNA. | | up regulated | |
| LOC641700 | PREDICTED: Homo sapiens hypothetical protein LOC641700 (LOC641700), mRNA. | | up regulated | |
| CTSK | Homo sapiens cathepsin K (CTSK), mRNA. | | up regulated | |
| UNQ5783 | Homo sapiens chromosome 17 open reading frame 87 (C17orf87), mRNA. | | up regulated | |
| COL8A1 | Homo sapiens collagen, type VIII, alpha 1 (COL8A1), transcript variant 1, mRNA. | | up regulated | |
| PDGFRB | Homo sapiens platelet-derived growth factor receptor, beta polypeptide (PDGFRB), mRNA. | | up regulated | |
| STC1 | Homo sapiens stanniocalcin 1 (STC1), mRNA. | | up regulated | |
| LUM | Homo sapiens lumican (LUM), mRNA. | | up regulated | |
| VMD2 | Homo sapiens vitelliform macular dystrophy 2 (Best disease, bestrophin) (VMD2), mRNA. | | up regulated | |
| CPVL | Homo sapiens carboxypeptidase, vitellogenic-like (CPVL), transcript variant 1, mRNA. | | up regulated | |
| HLA-DRB4 | Homo sapiens major histocompatibility complex, class II, DR beta 4 (HLA-DRB4), mRNA. | | up regulated | |
| COL1A2 | Homo sapiens collagen, type I, alpha 2 (COL1A2), mRNA. | | up regulated | |
| IGFBP2 | Homo sapiens insulin-like growth factor binding protein 2, 36kDa (IGFBP2), mRNA. | | up regulated | |
| NR2F1 | Homo sapiens nuclear receptor subfamily 2, group F, member 1 (NR2F1), mRNA. | | up regulated | |
| JSRP1 | Homo sapiens junctional sarcoplasmic reticulum protein 1 (JSRP1), mRNA. | | up regulated | |
| GPR4 | Homo sapiens G protein-coupled receptor 4 (GPR4), mRNA. | | up regulated | |
| PLEK | Homo sapiens pleckstrin (PLEK), mRNA. | | up regulated | |
| FZD2 | Homo sapiens frizzled homolog 2 (Drosophila) (FZD2), mRNA. | | up regulated | |
| C1QA | Homo sapiens complement component 1, q subcomponent, alpha polypeptide (C1QA), mRNA. | | up regulated | |
| C9ORF109 | PREDICTED: Homo sapiens chromosome 9 open reading frame 109 (C9orf109), mRNA. | | up regulated | |
| C1QB | Homo sapiens complement component 1, q subcomponent, beta polypeptide (C1QB), mRNA. | | up regulated | |
| IGFBP5 | Homo sapiens insulin-like growth factor binding protein 5 (IGFBP5), mRNA. | | up regulated | |
| WARS | Homo sapiens tryptophanyl-tRNA synthetase (WARS), transcript variant 1, mRNA. | | up regulated | |
| LOC651751 | PREDICTED: Homo sapiens similar to Ig kappa chain V-II region RPMI 6410 precursor (LOC651751), mRNA. | | up regulated | |
| CMKLR1 | Homo sapiens chemokine-like receptor 1 (CMKLR1), mRNA. | | up regulated | |
| CLEC10A | Homo sapiens C-type lectin domain family 10, member A (CLEC10A), transcript variant 2, mRNA. | | up regulated | |
| BHLHB5 | Homo sapiens basic helix-loop-helix domain containing, class B, 5 (BHLHB5), mRNA. | | up regulated | |
| ALDH1L1 | Homo sapiens aldehyde dehydrogenase 1 family, member L1 (ALDH1L1), mRNA. | | up regulated | |
| VWF | Homo sapiens von Willebrand factor (VWF), mRNA. | | up regulated | |
| HOXB8 | Homo sapiens homeo box B8 (HOXB8), mRNA. | | up regulated | |
| IL8RBP | PREDICTED: Homo sapiens interleukin 8 receptor, beta pseudogene (IL8RBP), misc RNA. | | up regulated | |
| TMEM45A | Homo sapiens transmembrane protein 45A (TMEM45A), mRNA. | | up regulated | |
| DYSF | Homo sapiens dysferlin, limb girdle muscular dystrophy 2B (autosomal recessive) (DYSF), mRNA. | | up regulated | |
| CCL2 | Homo sapiens chemokine (C-C motif) ligand 2 (CCL2), mRNA. | | up regulated | |
| UBD | Homo sapiens ubiquitin D (UBD), mRNA. | | up regulated | |
| LOC349136 | Homo sapiens WD repeat domain 86 (WDR86), mRNA. | | up regulated | |
| IRAK3 | Homo sapiens interleukin-1 receptor-associated kinase 3 (IRAK3), mRNA. | | up regulated | |
| HS.72010 | RST24587 Athersys RAGE Library Homo sapiens cDNA, mRNA sequence | | up regulated | |
| GAL | Homo sapiens galanin (GAL), mRNA. | | up regulated | |
| CD33 | Homo sapiens CD33 molecule (CD33), mRNA. | | up regulated | |
| OLFML2B | Homo sapiens olfactomedin-like 2B (OLFML2B), mRNA. | | up regulated | |
| SRPX2 | Homo sapiens sushi-repeat-containing protein, X-linked 2 (SRPX2), mRNA. | | up regulated | |
| COL4A1 | Homo sapiens collagen, type IV, alpha 1 (COL4A1), mRNA. | | up regulated | |
| SLC3A1 | Homo sapiens solute carrier family 3 (cystine, dibasic and neutral amino acid transporters, activator of cystine, dibasic and neutral amino acid transport), member 1 (SLC3A1), mRNA. | | up regulated | |
| SPP1 | Homo sapiens secreted phosphoprotein 1 (osteopontin, bone sialoprotein I, early T-lymphocyte activation 1) (SPP1), transcript variant 1, mRNA. | | up regulated | |
| NOX4 | Homo sapiens NADPH oxidase 4 (NOX4), mRNA. | | up regulated | |
| FCER1G | Homo sapiens Fc fragment of IgE, high affinity I, receptor for; gamma polypeptide (FCER1G), mRNA. | | up regulated | |
| LAIR2 | Homo sapiens leukocyte-associated immunoglobulin-like receptor 2 (LAIR2), transcript variant 2, mRNA. | | up regulated | |
| CPT1C | Homo sapiens carnitine palmitoyltransferase 1C (CPT1C), mRNA. | | up regulated | |
| LILRA5 | Homo sapiens leukocyte immunoglobulin-like receptor, subfamily A (with TM domain), member 5 (LILRA5), transcript variant 1, mRNA. | | up regulated | |
| PILRA | Homo sapiens paired immunoglobin-like type 2 receptor alpha (PILRA), transcript variant 1, mRNA. | | up regulated | |
| BGN | Homo sapiens biglycan (BGN), mRNA. | | up regulated | |
| CD163 | Homo sapiens CD163 molecule (CD163), transcript variant 1, mRNA. | | up regulated | |
| HLA-DRB6 | Homo sapiens major histocompatibility complex, class II, DR beta 6 (pseudogene) (HLA-DRB6) on chromosome 6. | | up regulated | |
| CPXM | Homo sapiens carboxypeptidase X (M14 family), member 1 (CPXM1), mRNA. | | up regulated | |
| C1ORF162 | Homo sapiens chromosome 1 open reading frame 162 (C1orf162), mRNA. | | up regulated | |
| FCGR3B | Homo sapiens Fc fragment of IgG, low affinity IIIb, receptor (CD16b) (FCGR3B), mRNA. | | up regulated | |
| FNDC1 | Homo sapiens fibronectin type III domain containing 1 (FNDC1), mRNA. | | up regulated | |
| TCN1 | Homo sapiens transcobalamin I (vitamin B12 binding protein, R binder family) (TCN1), mRNA. | | up regulated | |
| VSIG4 | Homo sapiens V-set and immunoglobulin domain containing 4 (VSIG4), mRNA. | | up regulated | |
| LOC649923 | PREDICTED: Homo sapiens similar to Ig gamma-2 chain C region (LOC649923), mRNA. | | up regulated | |
| PROK2 | Homo sapiens prokineticin 2 (PROK2), mRNA. | | up regulated | |
| CSF3R | Homo sapiens colony stimulating factor 3 receptor (granulocyte) (CSF3R), transcript variant 4, mRNA. | | up regulated | |
| NFE2 | Homo sapiens nuclear factor (erythroid-derived 2), 45kDa (NFE2), mRNA. | | up regulated | |
| FOXQ1 | Homo sapiens forkhead box Q1 (FOXQ1), mRNA. | | up regulated | |
| FCN1 | Homo sapiens ficolin (collagen/fibrinogen domain containing) 1 (FCN1), mRNA. | | up regulated | |
| CLEC12A | Homo sapiens C-type lectin domain family 12, member A (CLEC12A), transcript variant 3, mRNA. | | up regulated | |
| HK3 | Homo sapiens hexokinase 3 (white cell) (HK3), nuclear gene encoding mitochondrial protein, mRNA. | | up regulated | |
| NPTX2 | Homo sapiens neuronal pentraxin II (NPTX2), mRNA. | | up regulated | |
| HAS3 | Homo sapiens hyaluronan synthase 3 (HAS3), transcript variant 1, mRNA. | | up regulated | |
| S100A8 | Homo sapiens S100 calcium binding protein A8 (S100A8), mRNA. | | up regulated | |
| CCL8 | Homo sapiens chemokine (C-C motif) ligand 8 (CCL8), mRNA. | | up regulated | |
| MMP7 | Homo sapiens matrix metallopeptidase 7 (matrilysin, uterine) (MMP7), mRNA. | | up regulated | |
| MCEMP1 | Homo sapiens mast cell-expressed membrane protein 1 (MCEMP1), mRNA. | | up regulated | |
| NR1H4 | Homo sapiens nuclear receptor subfamily 1, group H, member 4 (NR1H4), mRNA. | | up regulated | |
| ALDOB | Homo sapiens aldolase B, fructose-bisphosphate (ALDOB), mRNA. | | up regulated | |
| DEFA5 | Homo sapiens defensin, alpha 5, Paneth cell-specific (DEFA5), mRNA. | | up regulated | |
| REG1A | Homo sapiens regenerating islet-derived 1 alpha (pancreatic stone protein, pancreatic thread protein) (REG1A), mRNA. | | up regulated | |
